# TNFα signaling in radiation-induced chronic bowel dysfunction suggests therapeutic potential for IBD biologics

**DOI:** 10.1186/s10020-026-01441-4

**Published:** 2026-04-09

**Authors:** Sravani Devarakonda, Piyush Patel, Amelie Toft Morén, Karin Bergmark, Mohammad Bamfarahnak, Patrik A. Buske, Yueling Peng, Annika Thorsell, Yuan Li, Henrik Fagman, Jennifer Fransson, Lisen Heden, Karin Gustafsson, Changlian Zhu, Per Hedenström, Eduardo J. Villablanca, Cecilia Bull

**Affiliations:** 1 Division of Clinical Cancer Epidemiology, Department of Oncology, Institute of Clinical Sciences, Sahlgrenska Academy, University of Gothenburg, Gothenburg, Sweden; 2 Center for Brain Repair and Rehabilitation, Institute of Neuroscience and Physiology, Sahlgrenska Academy, University of Gothenburg, Gothenburg, Sweden; 3 Proteomics Core Facility, Sahlgrenska Academy, University of Gothenburg, Gothenburg, Sweden; 4 Science for Life Laboratory, National Bioinformatics Infrastructure Sweden (NBIS), Lund University, Lund, Sweden; 5 Department of Immunotechnology, Lund University, Medicon Village, Lund, Sweden; 6 Department of Laboratory Medicine, Institute of Biomedicine, Sahlgrenska Academy, University of Gothenburg and Department of Clinical Pathology, Sahlgrenska University Hospital, Gothenburg, Sweden; 7 Division of Immunology and Respiratory Medicine, Department of Medicine, Karolinska Institute and University Hospital, Solna, Stockholm, Sweden; 8 Clinical Immunology and Transfusion Medicine, Karolinska University Hospital, Stockholm, Sweden; 9 Pelvic Cancer Rehabilitation, Sahlgrenska University Hospital, Gothenburg, Sweden; 10 Department of Molecular and Clinical Medicine, Institute of Medicine, Sahlgrenska Academy, University of Gothenburg and Department of Gastroenterology and Hepatology, Sahlgrenska University Hospital, Gothenburg, Sweden

**Keywords:** Pelvic radiotherapy, Chronic inflammation, Inflammatory bowel disease, Tumor necrosis factor-alpha, Enteroendocrine cells, Omics, Biologics

## Abstract

**Background:**

Pelvic radiation disease (PRD) arises from normal tissue damage following pelvic radiotherapy and often manifests as bowel dysfunction. Chronic low-grade inflammation is observed in the irradiated mucosa, but its clinical significance remains unclear, as broad-spectrum anti-inflammatory agents show limited efficacy for PRD-related symptoms. However, the potential of targeted biologics used in inflammatory bowel disease (IBD) has not been evaluated, and the chronic inflammatory profile of irradiated mucosa remains undefined. This study sought to evaluate the potential therapeutic utility of IBD biologics by characterizing chronic features of radiation-induced mucosal pathophysiology, with emphasis on low-grade inflammation.

**Methods:**

We performed mRNA sequencing and quantitative mass spectrometry on colorectal mucosal biopsies from pelvic cancer survivors (n = 27), collected 3–20 years post-radiotherapy, and from non-irradiated controls (n = 4). Gene and protein expression were compared across regions with high, low, or no prior radiation exposure. mRNA datasets from Crohn’s disease (n = 127), ulcerative colitis (n = 74), and healthy controls (n = 50) were used for comparative analysis and to identify shared treatment targets. Key findings were validated using complementary techniques and correlated with patient-reported symptoms.

**Results:**

Principal Component Analysis (PCA) confirmed dataset comparability and validated low-dose biopsies from PRD patients as suitable internal controls. In mucosa previously exposed to high-dose radiation, central metabolic programs such as heme metabolism, fatty acid metabolism, oxidative phosphorylation, and glycolysis were permanently suppressed, whereas inflammatory and repair pathways were enriched, including angiogenesis, epithelial–mesenchymal transition, DNA repair, cell proliferation, and TNF-α signaling via NF-κB, an important therapeutic target in IBD. Notably, enteroendocrine cells emerged as a source of TNF-α signaling in PRD. Increased IL-8 expression was also observed in PRD mucosa, along with elevated leukocyte-trafficking molecules targeted by anti-integrin therapies. In contrast, there was no or minimal evidence for JAK-STAT signaling, which is downstream of multiple cytokines implicated in IBD-associated inflammation. Individuals with severe bowel symptoms exhibited elevated expression of antigen presentation genes, suggesting a link between persisting immune activation and clinical manifestations.

**Conclusions:**

Years after pelvic radiotherapy, the colorectal mucosa displays a TNF-α–dominated inflammatory–regenerative signature and widespread metabolic suppression. Our findings support further exploration of IBD biologics, particularly TNF-α inhibitors and integrin inhibitors, as potential therapeutic candidates in PRD.

**Graphical Abstract:**

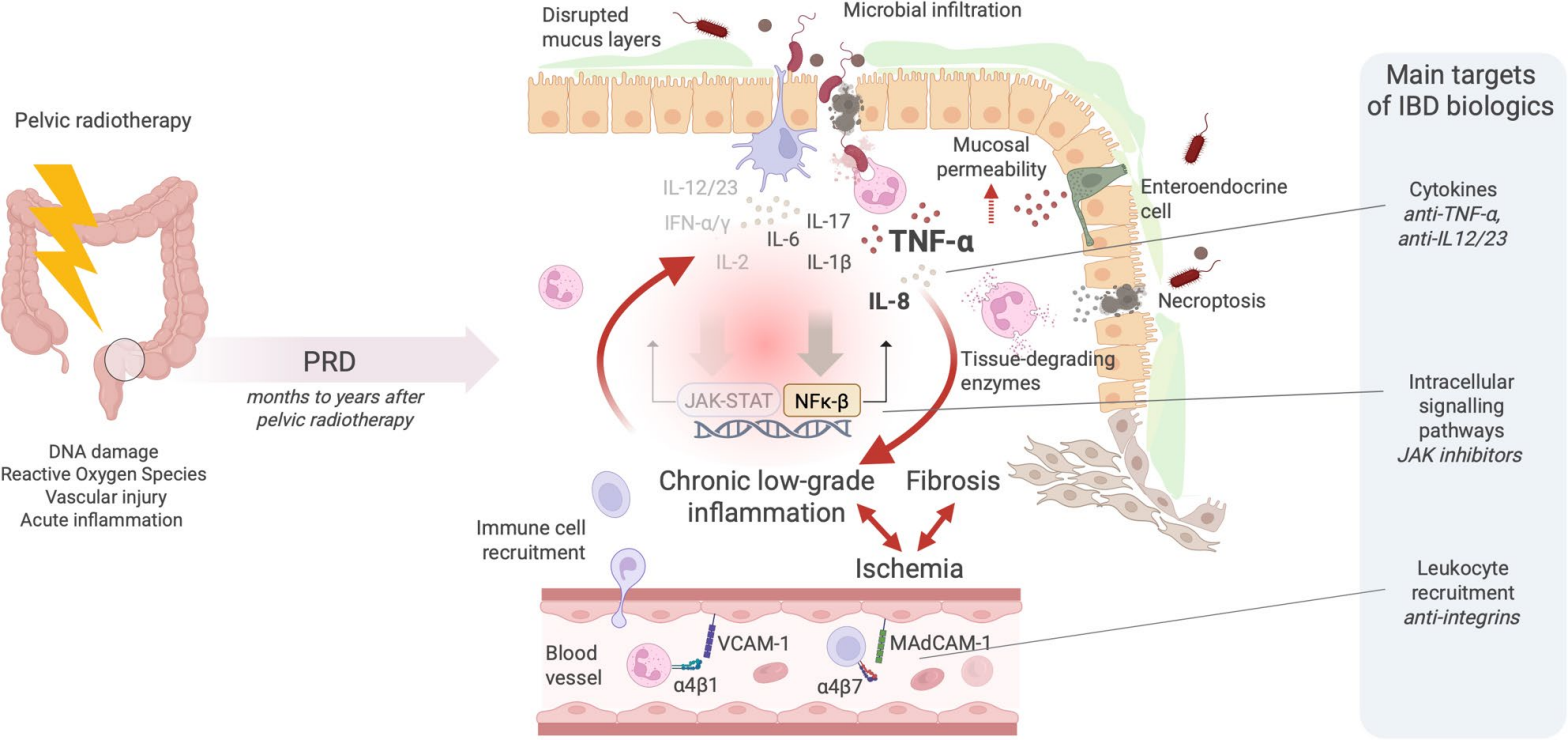

**Supplementary Information:**

The online version contains supplementary material available at 10.1186/s10020-026-01441-4.

## Background

Radiation-induced chronic bowel dysfunction following pelvic radiotherapy for malignancies in the pelvic area (e.g., prostate, rectal, anal, or gynecological cancers) is a growing clinical challenge due to increasing cancer survival rates and longer life expectancies. The condition manifests with debilitating symptoms such as diarrhea, tenesmus, fecal incontinence, bloating, and rectal bleeding and is referred to as pelvic radiation disease, “PRD”, an umbrella term for symptoms arising from pelvic organs exposed to radiation (Steineck et al. 2017; Andreyev et al. 2010). The majority of irradiated pelvic cancer survivors is believed to experience some degree of PRD, with approximately 50% reporting a reduced quality of life (Andreyev et al. 2010).

Bowel-associated PRD has traditionally been attributed to intestinal fibrosis and ischemia, with no underlying inflammatory component (Mahmood et al. 2021; Morris and Haboubi 2015). More recently, we demonstrated that, in addition to causing ischemia and fibrosis, pelvic radiotherapy results in a permanently disrupted mucus barrier, bacterial infiltration, and chronic, low-grade mucosal inflammation with neutrophil engagement (Devarakonda et al. 2023). It remains unknown whether this chronic low-grade inflammation contributes to ischemia and fibrosis, or any of the symptoms experienced by irradiated pelvic cancer survivors. Compared to inflammatory bowel disease (IBD; *e.g.,* Crohn’s disease and ulcerative colitis), inflammation in PRD mucosa is very subtle, and immune cell infiltration is barely discernible in tissue sections. This may explain why the inflammatory component of PRD has not been properly recognized or characterized. Moreover, limited efficacy of broad-spectrum anti-inflammatory agents in treating PRD symptoms challenges the hypothesis that chronic low-grade inflammation plays a significant clinical role in the condition (Lawrie, et al., 2018). However, the potential benefit of targeted anti-inflammatory biologics, widely used in IBD, remains largely unexplored in PRD. Examples of these biologics include TNF-α (tumor necrosis factor-alpha) inhibitors, integrin inhibitors, interleukin-12 (IL-12)/IL-23 inhibitors, and janus kinase (JAK) inhibitors (Neurath 2024). We previously described a case of severe chronic PRD successfully treated with the TNF-α inhibitor infliximab (Toft Morén et al. 2023). To our knowledge, this remains the only documented case of its kind, although IBD biologics may have been used off label in other isolated instances that have not been reported.

The potent response described in our previous case report may be exceptional, and given the potential side effects of IBD biologics, a strong rationale for their use is needed. At present, the low-grade inflammation in PRD mucosa is poorly defined, and it is unclear which pharmacological mechanism, if any, is most likely to modulate it effectively. Furthermore, current evidence that targeting this inflammation leads to a clinically meaningful improvement in PRD symptoms is very limited. To begin addressing these uncertainties, we applied global, high-resolution multi-omics to characterize pathophysiological processes in the mucosa of PRD patients as late as 20 years after pelvic radiotherapy. Comparisons were made to IBD, where effective therapies exist, by analyzing publicly available mRNA datasets from biopsies of patients with Crohn’s disease, ulcerative colitis, and healthy controls. To link molecular profiles with clinical presentation, key findings were correlated with patient-reported outcomes obtained using validated clinimetric questionnaires. This approach revealed key similarities and differences between PRD and IBD, identified potential therapeutic targets, and advanced our understanding of chronic, low-grade mucosal inflammation following pelvic radiotherapy.

## Methods

### Study design

**Fig. 1:**
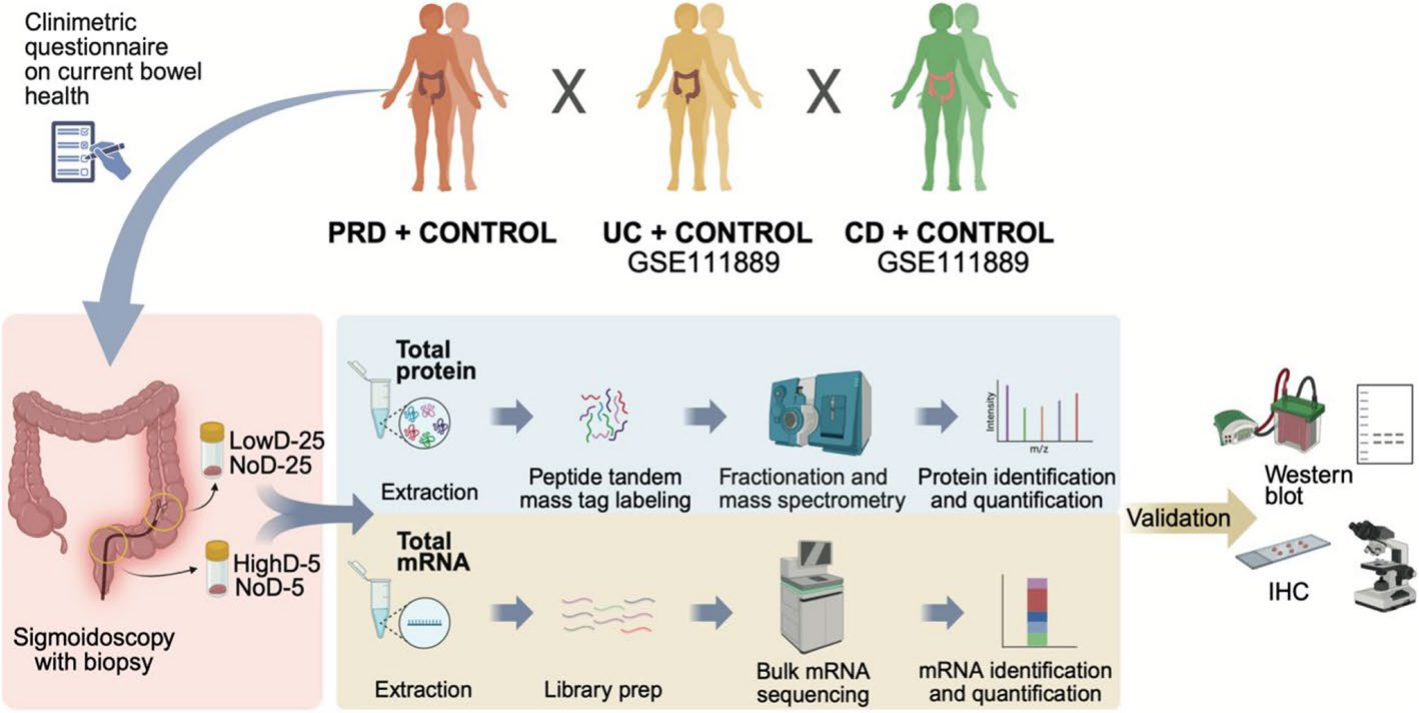
Study design. The PRD study participants (n = 27) were chosen from electronic medical records at a pelvic cancer rehabilitation clinic at the hospital. Biopsies were taken at different locations; ∼5 cm (high radiation dose area; “highD-5”) and ∼25 cm (low radiation dose area; “lowD-25”) from the anal verge using sigmoidoscopy. Non-irradiated controls (n = 4) were biopsied in an identical manner at ∼5 cm (no radiation dose area; "noD-5") and ∼25 cm (no radiation dose area; "noD-25"). Protein and mRNA were extracted and analyzed with tandem mass tag mass spectrometry and RNA sequencing, respectively. The IBD datasets on mucosal biopsies from UC (n = 74), CD (n = 127) and healthy controls (n = 50) were retrieved from the GEO repository (GSE111889). Western blot analysis and immunohistochemistry were used for validation

The study design is shown in Fig. 1. Eligible participants (PRD; n = 27) were identified through electronic medical records at the Pelvic Cancer Rehabilitation Unit at Sahlgrenska University Hospital, a women-focused clinic. This enabled the inclusion of well-characterized participants with extensive longitudinal clinical data. Biopsies were collected from two locations: 5 cm from the anal verge, where tissues generally receive a high off-target radiation dose during radiotherapy for pelvic cancers (high-dose biopsy; “highD-5”), and 25 cm from the anal verge, where there is usually little to no radiation exposure (low-dose biopsy; “lowD-25”, serving as internal control). Non-irradiated controls (n = 4) were identified via the endoscopy department at Sahlgrenska University Hospital and biopsied in an identical manner (“noD-5” and “noD-25”). Biopsies were collected in either RNALater or Histofix or were snap-frozen depending on the analysis. The biopsy procedure and inclusion/exclusion criteria are detailed in a previous publication (Devarakonda et al. 2023).

### Symptom documentation

The participants completed a clinimetric questionnaire on current bowel health. Three questions targeting urgency, loose stools, and tenesmus were selected for clustering the study participants with regards to their symptom intensity (see Additional Material 2 for questions and scoring). The selected questions targeted particularly debilitating symptoms (Baloch et al. 2022) that may be associated with inflammation, as other inflammatory conditions produce similar symptoms (Olesen et al. 2004; DuPont 2014).

### RNAseq data analysis

RNA was isolated and sequenced from biopsies collected in RNALater (PRD; n = 21, non-irradiated controls; n = 4). The procedures are described in a previous publication (Devarakonda et al. 2023), together with the counts and differential expression results which were incorporated in this analysis. Briefly, gene expression counts were generated from sequencing data using kallisto (Bray et al. 2016) and paired differential expression analysis was performed between low-dose and high-dose biopsies (“lowD-25” versus “highD-5”) using edgeR (Robinson et al. 2010), with likelihood ratio test to assess significance.

Gene expression counts from ulcerative colitis (“UC”; n = 74) and Crohn’s disease (“CD”; n = 127) patient samples and the corresponding healthy controls (“HC”; n = 50) were downloaded from Gene Expression Omnibus (GEO) at accession number GSE111889 (Lloyd-Price et al. 2019). Differential expression analysis was performed to compare UC vs HC and CD vs HC using edgeR. Significance was assessed in each comparison using quasi-likelihood F-tests.

The Benjamini-Hochberg procedure was used to correct for multiple tests. To perform principal component analysis across studies, counts were normalized using the cpm() function from edgeR and log-transformed (log2(x + 1)), and batch effects were removed using the removeBatchEffect() from edgeR using study and intestinal site as batch variables.

### Mass spectrometry-based proteomic analysis using tandem mass tags (TMT)

To identify instances where mRNA expression was confirmed at the protein level, we matched the differentially expressed mRNA with protein expression data retrieved from TMT mass spectrometry analysis of a subset of the study participants (n = 10). These individuals were not selected based on any clinical or demographic criteria but were included in order of enrollment. The sample processing for mass spectrometry and proteomics data analysis was performed as previously described (Devarakonda et al. 2023).

### Functional enrichment analysis

To better understand the biological roles of the significantly differentially expressed genes/proteins identified from the RNA-seq and proteomics data, Gene Set Enrichment Analysis (GSEA) was performed on ranked lists of differentially abundant genes/proteins (ranked by log2 fold change between the control and disease groups). This analysis was conducted using the gseKEGG() and GSEA() functions from the *clusterProfiler* R package (v4.6.2). For the GSEA() function, the Hallmark gene set collection from the Molecular Signatures Database (MSigDB; *msigdbr*v7.5.1) was used and only pathways with an adjusted p-value (p.adjust) ≤ 0.1 and a gene set size (setSize) ≥ 10 were selected for plotting. For the gseKEGG() function, only KEGG pathways with p.adjust ≤ 0.001 and setSize ≥ 10 were retained for visualization. GSEA was applied independently to each dataset, and the results were then combined into a single summary plot using the ggplot() function from the *ggplot2* R package (v3.4.1; (Wickham 2010)). In addition, we combined the univariate linear model (ULM) and multivariate linear model (MLM) from the decoupleR v.2.4.0 (Schubert et al. 2018) (Badia et al. 2022) package and the *PROGENy* pathway collection v1.20.0 (Schubert et al. 2018) to calculate a consensus score for estimating signaling pathway activities. These estimates were based on the log2 fold changes in gene/protein abundance between the control and disease groups. In PROGENy, only significant genes (p < 0.05) per pathway were selected.

For each pathway of interest, a heatmap was generated based on the log2 fold change of abundance between the control and disease groups for either all, or only significantly differentiated (FDR < 0.001 for transcriptomics data and *qval* < 0.05 for proteomics data; |log2FC|> 0.5), leading edge genes using the ggplot() function as implemented in the R package ggplot2 v.3.4.1. (Wickham 2010).

For the KEGG pathways of interest, log2 fold changes (logFC) in gene/protein abundance between the control and disease groups were visualized on KEGG pathway maps using the pathview() function from the *pathview* R package (v1.38.0). Genes/proteins with an absolute logFC less than 1 (|logFC|< 1) were excluded from mapping.

### Western blot analysis 

TNF-α protein expression in the mucosa was semi-quantified in subjects from whom protein samples remained after the TMT mass spectrometry analysis (PRD; n = 9, non-irradiated controls; n = 3). These samples were mixed with 4 × NuPAGE LDS buffer (NP0007, ThermoFisher Scientific Inc., Waltham, MA), 10 × sample reducing agent (NP0004, ThermoFisher Scientific Inc.), and Millipore water, adjusting the protein concentration as required, and incubated on a preheated heat block at 95 °C for 5 min. Each well of a 10% NuPAGE Bis–Tris gel (Invitrogen, Carlsbad, CA) was loaded with a total of 30 µg of protein. Electrophoresis was carried out for 40 min. Separated proteins were wet transferred to a nitrocellulose membrane (Bio-Rad Laboratories, Inc., Hercules, CA). The transfer was completed in 60 min. The membranes were washed in TBST and incubated in 5% non-fat milk in TBST buffer (20 mM Tris, 150 mM NaCl, and 0.1% Tween 20, pH 7.6) for 1 h on a shaker. Recombinant monoclonal rabbit anti-TNF-α (ERP19147, 1:1000 dilution, Abcam Limited, Cambridge, UK) and mouse monoclonal anti-human glyceraldehyde-3-phosphate dehydrogenase (GAPDH, clone 4G5, 1:1000 dilution, Bio-Rad Laboratories, Inc.) were added to the blocking buffer and left on a shaker overnight at 4 °C. The membranes were then washed in TBST and incubated with peroxidase-labeled goat anti-rabbit IgG antibody (PI-1000, 1:2000 dilution, Vector Laboratories, Newark, CA) or peroxidase-labeled horse anti-mouse IgG antibody (PI-2000, 1:4000 dilution, Vector Laboratories) for 1 h. Finally, the membranes underwent another three washes in 1X TBST and were placed between filter papers to air dry for a minimum of 2 h. The blots were visualized using the SuperSignal™ West Femto Chemiluminescent Substrate (cat. nr. 34,094, ThermoFisher Scientific Inc.) and a LAS 3000 cooled CCD camera (Fujifilm, Tokyo, Japan) and quantified using ImageJ software (National Institutes of Health, Bethesda, MD). Only samples with enough protein for the detection of TNF-α and a clear GAPDH band indicating proper loading and transfer were analyzed (PRD; n = 6, non-irradiated controls; n = 3).

### Immunohistochemistry

Biopsies were transferred to a buffered formaldehyde solution (Histofix; Histolab Products AB, Askim, Sweden) and embedded in paraffin blocks. Blocks from high-dose biopsies (n = 3) were sectioned in a 1:3 series, with three 4-μm-thick tissue sections placed on each slide. Two consecutive slides were used for immunohistochemistry with TNF-α and CgA, allowing two adjacent tissue sections to be labeled with each antibody. TNF-α immunostaining (primary antibody cat. no. LS-B7268, LifeSpan BioSciences, Inc. Seattle, WA) was carried out as described in Psaltis et al., (Psaltis et al. 2021) with the Novolink Polymer Detection System (cat. no. RE7140-K Leica Microsystems Gmbh, Wetzlar, Germany) according to the manufacturer’s protocol. Immunohistochemical staining of CgA (mouse monoclonal; clone LK2H10; dilution 1:6000, Sigma Aldrich, Burlington, MA, USA) was performed in a Dako Autostainer Link using the EnVision™ FLEX detection system according to the manufacturer's instructions (Agilent Technologies, Glostrup, Denmark).

### Symptom analysis

To identify patient subgroups based on the symptoms, we performed unsupervised k-means clustering using the scores retrieved from three questions about urgency, loose stools and repeated defecation (see Additional Material 1 for details). Based on the scree plot and the elbow method, three distinct clusters were identified: high symptom intensity, mixed symptom intensity, and low symptom intensity. The high symptom intensity cluster and the low symptom intensity cluster were selected for differential gene expression analysis.

For differential analysis between the high- and low-intensity symptom clusters, only genes with more than five raw counts in at least three samples were retained. We then applied the TMM normalization on the filtered count data to get log2 counts per million (logCPM) reads using the calcNormFactors() and voom() functions, which are from edgeR (version 3.40.2) and limma (version 3.54.2), respectively. (Ritchie et al. 2015; Law et al. 2014) Voom() also modeled the mean–variance relationship across genes and estimated precision weights for each observation so that genes with more consistent data get higher weights. Differential expression analysis was conducted using the lmFit() function, incorporating the logCPM data and the precision weights, followed by the calculation of the moderated statistical significance of the differential expression results through borrowing information across genes to improve variance estimates using the eBayes() function. Both lmFit() and eBayes() are from the limma package. To elucidate the biological significance of the differentially expressed genes between the high- and low-intensity symptom clusters, GSEA was performed using the gseKEGG() function. The results were visualized with a dot plot, displaying only pathways with an adjusted p-value (p.adj) < 0.001. For pathways of particular interest, the log fold change (logFC) values of the leading-edge genes were compiled and visualized in a heatmap using the pheatmap() function from the pheatmap package (version 1.0.12). Additionally, all genes from the TNF-α pathway present in the RNA-seq dataset were visualized in a separate heatmap using the same function.

## Results

### Study design and demographics

The study design is shown in Fig. 1 and described in the figure legend. The demographics of the study participants are shown in Table 1**.** All but one cancer survivor and two non-irradiated controls were female. The median age of the irradiated study participants at the time of biopsy collection was 66 years (ranging from 31–82 years) for the cancer survivors. The non-irradiated controls had a median age of 63.5 years (ranging from 47–88 years). The time in years between radiotherapy and biopsy collection ranged from 3 to 20 years, with the median time being 5 years.

**Table 1:**
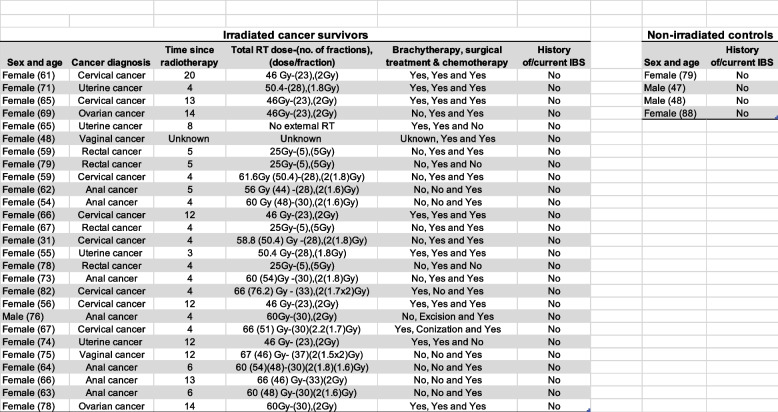
Demographics

### Similar gene expression profile in low-dose biopsies and control biopsies

**Fig. 2:**
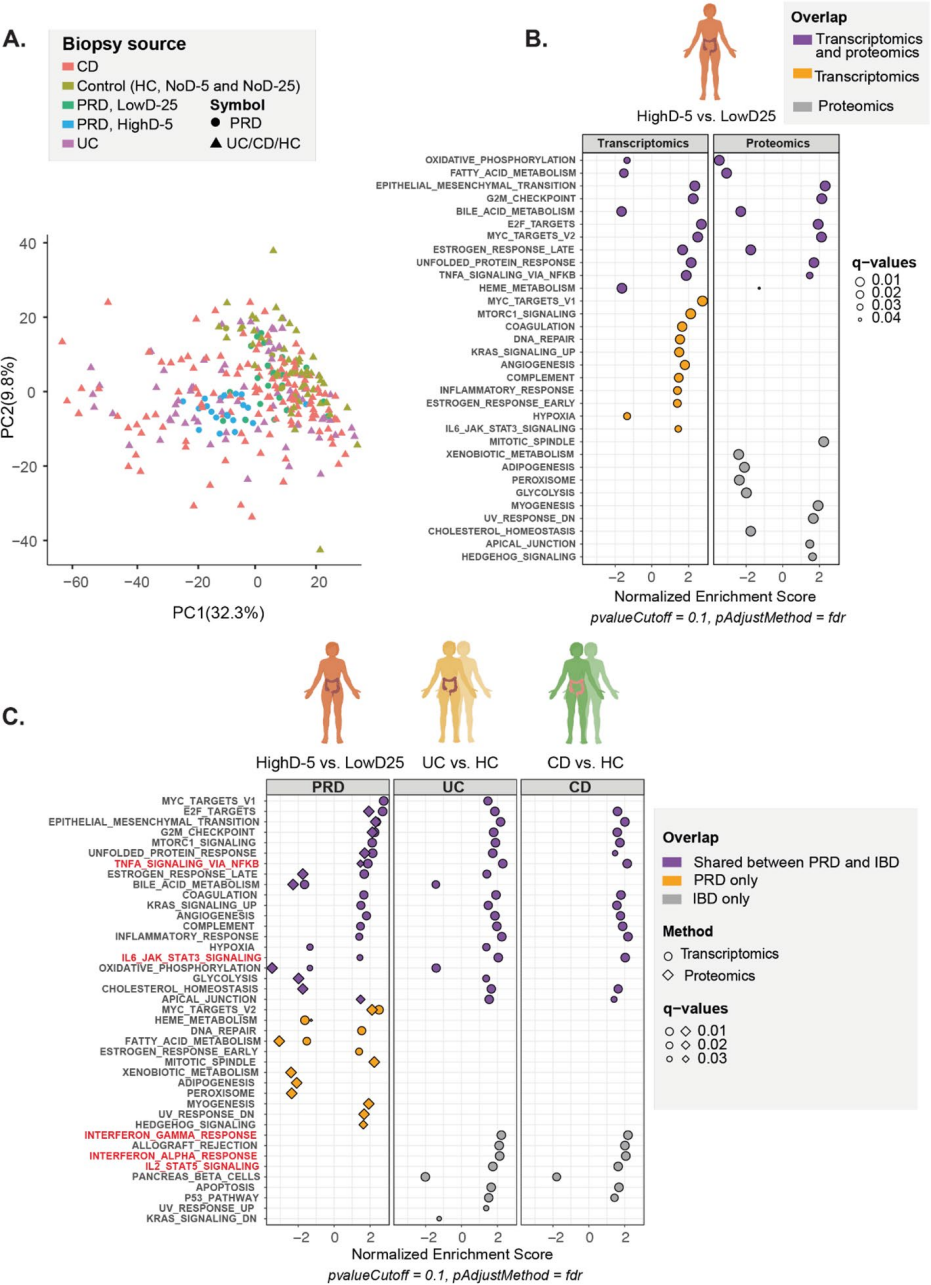
Differentially regulated biological processes and pathways in PRD and IBD. **A** Principal Component Analysis (PCA) on gene expression profiles. PCA included six datasets: "lowD-25" and "highD-5" biopsies from PRD patients (n = 21), "noD-25" and "noD-5" biopsies from non-irradiated controls (n = 4) as well as case and control biopsies from the GSE111889 (CD: n = 127; UC: n = 74; HC: n = 50) dataset. The control samples from all conditions clustered together (green color keys), indicating consistency across datasets. **B**. Activated or suppressed biological processes in the colorectal mucosa following pelvic radiotherapy. A positive value indicates enrichment in the high-dose biopsies compared to the low-dose biopsies. A negative value indicates a reduction. 32 biological processes were differentially regulated either in both the proteomic and the transcriptomic data sets (purple), the transcriptomic data set exclusively (orange) or the proteomic data set exclusively (gray). Symbol size = q-value. **C**. Comparison of activated or suppressed pathways in PRD and IBD. Regulated pathways identified from mRNA expression in PRD (lowD-25 vs. highD-5) and the IBD data sets (HC vs. UC, CD) are depicted with circles. Diamonds depict proteomic data. The x-axis within each of the three panels (PRD, UC and CD) shows the normalized enrichment score. A positive value represents an enriched pathway in case biopsy versus controls, and a negative value represents a suppressed pathway. 20 biological pathways were differentially regulated between case biopsies and controls in PRD and UC and/or CD (purple symbols). 12 pathways were unique for PRD (orange symbols), and 9 were only identified in UC and/or CD (grey symbols). Pathways known to be involved in the IBD inflammatory response are highlighted in red. Symbol size = q-value

To ensure that the low-dose biopsies were suitable as internal controls and to verify that our mRNA datasets were comparable to the datasets from the public database, we used Principal Component Analysis (PCA). We plotted gene expression profiles of case biopsies from the three conditions (“highD-5”, “UC” and “CD” biopsies), and their control biopsies (PRD internal control biopsies “lowD-25”, PRD external control biopsies “noD-25” and “noD-5”, and healthy control biopsies “HC” from UC and CD data sets). On the PC1–PC2 plot (explaining 32.3% and 9.8% of variance, respectively), the control sets from all three disease conditions clustered closely together, exhibiting a tight joint distribution, supporting that the low-dose biopsies were suitable as internal controls, and that the datasets were compatible (Fig. 2A). These control samples partially overlapped with the conditions, which displayed slightly broader dispersion along PC1.

### Pelvic radiotherapy permanently disrupts major mucosal biological processes

Our previous work identified inflammation-associated mRNAs and proteins that are altered in PRD mucosa (Devarakonda et al. 2023). Here, to explore permanently disrupted biological processes and pathways, we compared gene expression in the high-dose biopsies with gene expression in the low-dose biopsies (“highD-5” versus “lowD-25”) using GSEA-MSigDB Hallmark Pathways (Fig. 2B). GSEA-MSigDB Hallmark Pathways is a collection of 50 curated gene sets representing well-defined biological pathways (Liberzon et al. 2015). In parallel, to strengthen the biological interpretation, differentially expressed pathways from the transcriptomic analysis were compared with matched protein-level data. Eleven regulated pathways were identified in both the mRNA and protein data sets (purple color key). In all but one pathway – *“estrogen_response_late”* – the mRNA and protein expression pathways were regulated in the same direction (either enriched or suppressed). Notably, multiple metabolic pathways – *“bile_acid_metabolism”*, *“fatty_acid_metabolism”, “heme metabolism”* – and *“oxidative_phosphorylation”* – were suppressed in the high-dose biopsies compared to the low-dose biopsies. Conversely, the inflammation-related pathway *“TNFA_signaling_via_NFKB”* (nuclear factor kappa-light-chain-enhancer of activated B cells) and pathways involved in fibrosis and repair response were enriched, including “*epithelial_mesenchymal_transition*” and *“G2M checkpoint”.*

An additional 11 differentially regulated pathways between the high-dose biopsies and the low-dose biopsies were identified in the mRNA data sets only (orange color key). Some of the enriched pathways were *“inflammatory_response”, “IL6_JAK_STAT3_signalling”, “complement”, “angiogenesis”, “coagulation”,* and* “DNA_repair”.*

Ten regulated pathways were identified in the protein data sets only (gray color key). Several of these pathways related to metabolism and were suppressed in the high-dose biopsies compared to the low-dose biopsies, including *“xenobiotic metabolism”, “glycolysis”, “peroxisome”* and *“cholesterol_homeostasis”.* Enriched pathways included regenerative pathways such as *“mitotic_spindle”, “UV_response_dn”,* and *“Hedgehog_signaling”.*

### PRD mucosa displays chronic TNF-α activity but limited JAK-STAT and interferon signaling

Next, to identify similarities and differences in the regulation of biological processes and pathways among the three conditions, we analyzed MSigDB hallmark gene pathway activity in PRD (“highD-5” vs. “lowD-25”) and IBD (“UC” or “CD” vs. “HC”) (Fig. 2C). For PRD, we also matched the differentially expressed transcripts with corresponding protein expression data to validate our findings at the protein level. Twenty regulated pathways were identified in both PRD and IBD (purple color key). Twelve pathways were identified in PRD only (orange color key) and nine in IBD only (gray color key).

There was evidence supporting TNF-α activation through NF-κB *(“TNFA_signaling_via_NFKB”)* across all three disease conditions. Enrichment for this pathway was observed in both transcriptomic and proteomic data from PRD, thereby supporting the reliability of the finding. Several additional pathways exhibited similar regulation between all three conditions and were enriched in the case biopsies versus the control biopsies, including *“coagulation”, “angiogenesis”, “complement”, “inflammatory_response”, and “IL6_JAK_STAT3_signaling”.* However, compared to IBD, there was weak evidence of IL-6-JAK-STAT3 signaling pathway activity in PRD. Also, there was no evidence for signaling through interferons or IL-2-STAT5 *(“interferon_gamma_response”, “interferon_alpha_response”* and *“IL2_STAT5_signaling”)* in PRD.

Pathways that were exclusively identified in PRD and enriched in the high-dose biopsies included pathways involved in cell dynamics such as *“DNA_repair”, “myc_targets_v2 “, “hedgehog_signaling”* and *“mitotic spindle”*. Suppressed pathways included the metabolic pathways *“fatty_acid_metabolism”* and *“heme metabolism”*, among others.

Some pathways that were exclusively identified in IBD and enriched in the case biopsies were *“interferon_gamma_response”, “interferon_alpha _response”, IL2_STAT5_signaling”, “apoptosis”* and* “P53_pathway”.*

Analysis by GSEA and MSigDB Hallmark focuses on curated gene sets that represent a broad range of biological processes or pathological conditions. In contrast, gseKEGG  analysis identifies specific metabolic or signaling pathways from the KEGG database but does not account for the redundancy caused by genes that appear in multiple pathways (Liberzon et al. 2015). Supplementary Fig. 1 shows the result of a gseKEGG analysis of PRD, UC, and CD biopsies. *“IL-17 signaling pathway”* and *“TNF signaling pathway”* were enriched in all three conditions compared to respective controls*. “NF-kappa B signaling pathway”* and *“JAK-STAT signaling pathway”* were enriched in UC and CD but not identified in PRD. For the pathways identified at both the protein and mRNA levels (10 in total) the direction of regulation (enriched or suppressed) was consistent. As with the MSigDB analysis, several metabolic pathways were suppressed in PRD, while fibrotic and regenerative processes were enriched.

### PROGENy-based pathway inference supports GSEA-derived findings

**Fig. 3:**
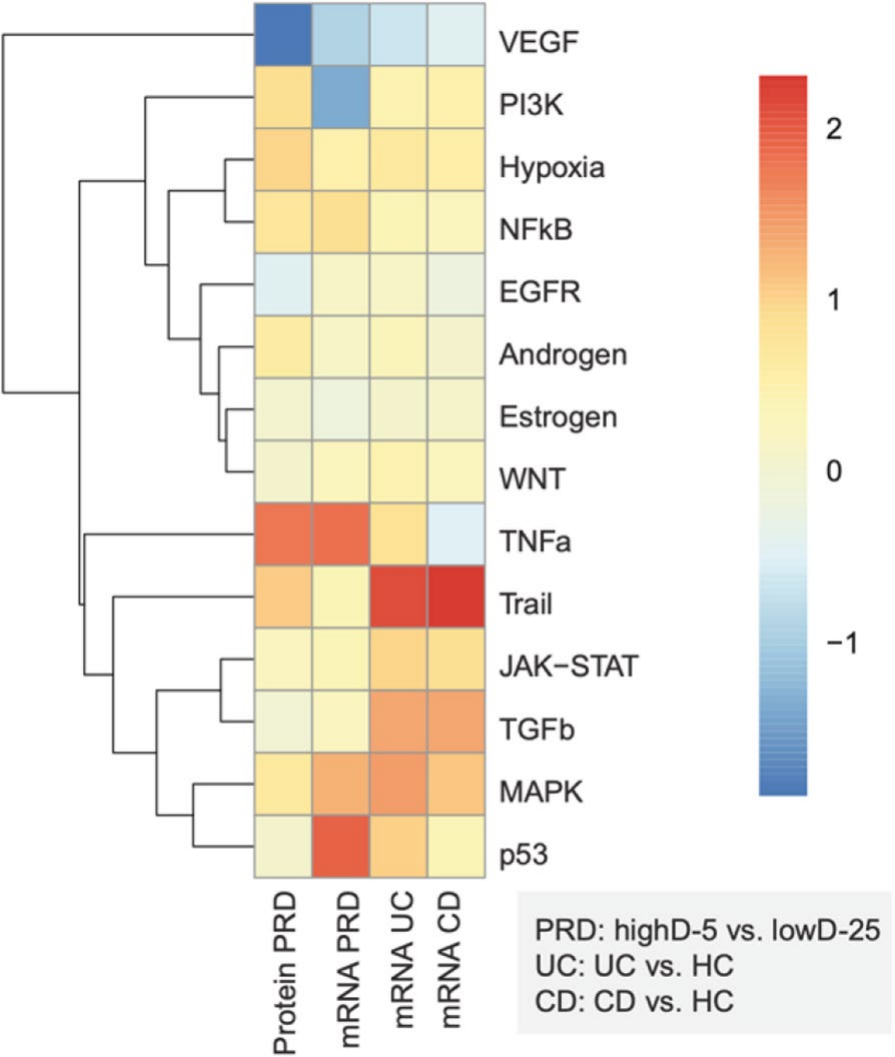
Biological activity of selected pathways in PRD and IBD. PROGENy heat map of pathway activity using PROGENy analysis of the PRD mRNA and protein data sets, and the mRNA data sets for UC and IBD versus HC. To infer biological activity from the data, a consensus score was generated across ULM and MLM in decoupleR. The color represents the biological activity score. Only genes deemed statistically significant (p < 0.05) per pathway were chosen for the analysis

In addition to the GSEA, we estimated the activity of PROGENy (Pathway RespOnsive GENes) signaling pathways in the three conditions using decoupleR **(**Fig. 3**).** Unlike KEGG and MsigDB-hallmark, which have annotated gene members for each gene set, PROGENy has responsive genes (with weights) for each of 14 signaling pathways (VEGF, PI3K, Hypoxia, NFkb, EGFR, Androgen, Estrogen, WNT, TNFa, Trail, JAK_STAT, TGFb, MAPK, and p53) (Schubert et al. 2018).

We observed relatively strong activation of the TNF-α pathway in our PRD dataset at both the mRNA and protein levels. In contrast, UC showed weaker TNF-α pathway activity, while CD exhibited reduced TNF-α pathway activity relative to control. In line with the GSEA results, JAK-STAT signaling was indicated in IBD but absent or weak in PRD. However, since the datasets originate from different sources (our own study versus the GEO repository database) and are thereby subject to unknown clinical and experimental variables, direct comparison of pathway scores across conditions should be avoided. Moreover, depending on signaling dynamics in a given condition, one method (GSEA or PROGENy) may capture activity better than the other.

In PRD, most of the regulated pathways identified at both the mRNA and protein levels were regulated in the same direction; however, translational correlation was not always consistent. For example, while mRNA expression patterns for the PI3K pathway suggested inhibition in PRD, the protein data indicated mild activation. Due to the lack of protein data for UC and CD, we were unable to assess the translational correlation of TNF-α pathway activity in these conditions.

### Evidence for TNF-α signaling through NF-κB in PRD

**Fig. 4:**
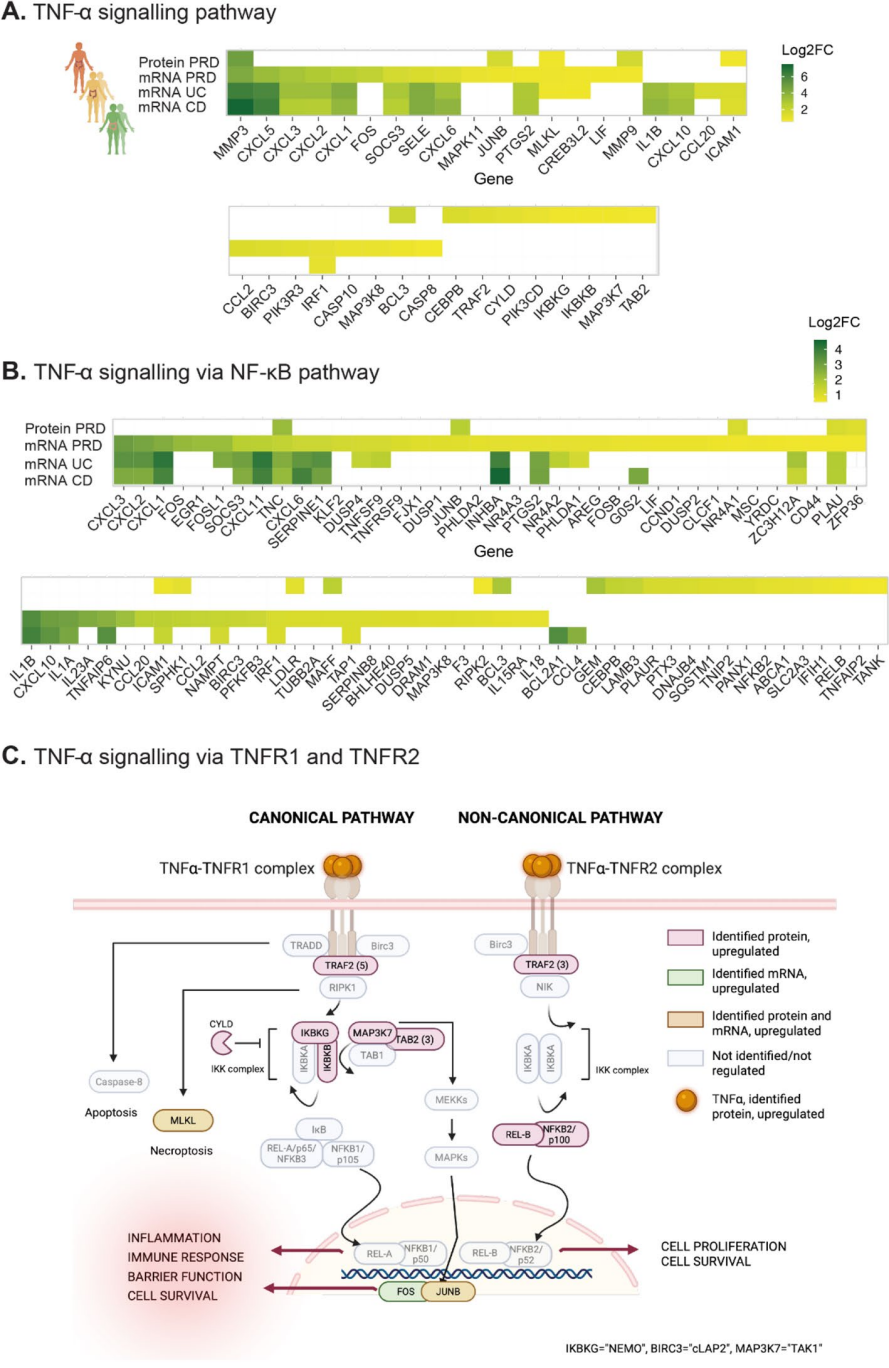
In-depth analysis of TNF-α signaling. The change in protein and/or mRNA expression levels of individual genes in the **A**. TNF-α signaling pathway and **B**. TNF-α signaling via NF-κB pathway were assessed between the three conditions and their respective controls. The top row represents proteomic data. Only the leading-edge genes that were significantly differentially expressed in at least one group are shown. [FDR < 0.001 for transcriptomics and 0.05 for proteomics, |log2FC|> 0.5]). White fields: NA/non-significant. **C.** Illustration of TNF-α-TNFR signaling via the canonical and non-canonical pathways. Key proteins and mRNA identified as increased in the high-dose irradiated mucosa are highlighted

We have previously reported on altered gene expression related to inflammation in the PRD cohort (Devarakonda et al. 2023). To further characterize gene expression in pathways targeted by IBD biologics, we conducted GSEA on ranked lists of differentially abundant mRNAs and proteins in selected pathways. Only leading-edge genes significantly regulated in at least one group are shown (Fig. 4A and 4, and Supplementary Fig. 2A-C). Several mRNAs involved in the TNF-α pathway, including the chemokine (C-X-C motif) ligands (CXCL) 1, CXCL2, CXCL3, CXCL5, CXCL6, and matrix metalloproteinase (MMP)−3 were upregulated across all conditions. MMP-3 showed multifold protein-level upregulation in PRD. Leading-edge genes and proteins in the IL-17 pathway, IL-6-JAK-STAT3 and JAK-STAT pathways are shown in Supplementary Fig. 2 A and B.

KEGG Pathview enables visual comparison of gene expression across the conditions. Comparisons in the TNF-α and IL-17 pathways between PRD and IBD are shown in Supplementary Figs. 3A–C and 4A–C, respectively. Differentially expressed cytokine and receptor mRNAs are shown in Supplementary Fig. 5A–C.

### TNF-α activates both canonical and non-canonical pathways in PRD

TNF-α promotes NF-κB activity via TNFR1 and the canonical pathway or TNFR2 and the non-canonical pathway, triggering distinct cellular responses (Souza et al. 2023) (Taniguchi and Karin 2018). We outlined key signaling molecules in the canonical and non-canonical pathway and highlighted the differentially expressed genes and proteins in our PRD cohort (Fig. 4C). We found that TNF-α appears to activate both the canonical and the non-canonical pathways in the high-dose biopsies. Of note, the necroptosis protein MLKL (mixed-lineage kinase domain-like protein) was upregulated at both mRNA and protein level, indicating persistent necroptosis in the intestinal mucosa following radiation exposure.

### Validation of chronic TNF-α expression in irradiated mucosal tissue

**Fig. 5:**
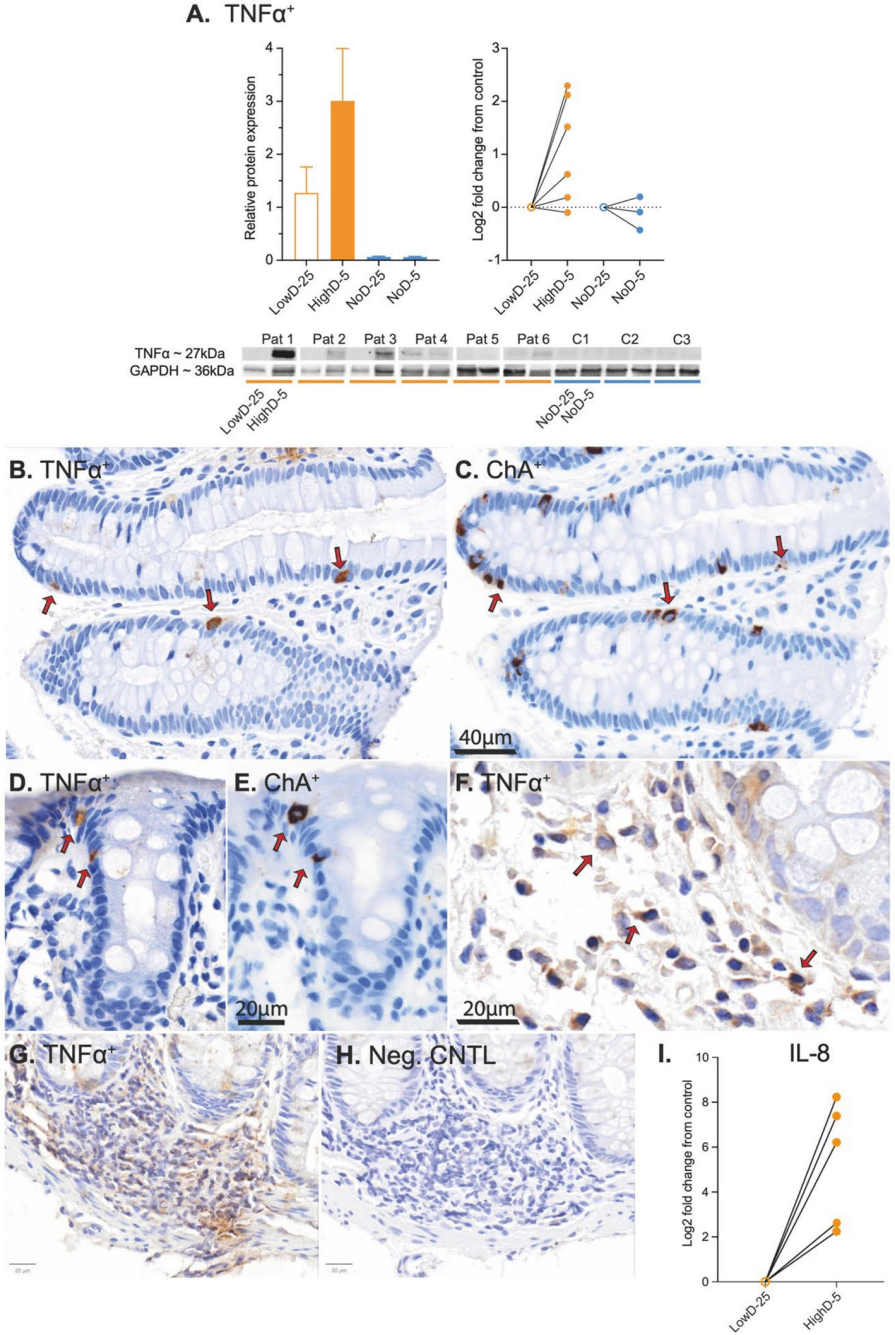
Source of TNF-α expression in PRD mucosa. **A**. Comparison of TNF-α expression, normalized against GAPDH, and fold changes in TNF-α expression between PRD case biopsies and their internal controls (lowD-25 vs. highD-5, and noD-25 vs. noD-5). Lines connect the biopsy pairs. **B**. Immunohistochemistry shows that cells in the crypt lining contribute to TNF-α in the tissue (red arrows, DAB/hematoxylin).** C**. Staining of adjacent sections indicates that the TNF-α^+^ cells in the crypt lining are CgA^+^ enteroendocrine cells (red arrows, DAB/hematoxylin). **D** and **E**. Colocalization of TNF-α and CgA (red arrows).** F**. TNF-α^+^ cells in the lamina propria (red arrows). **G.** Lymphoid follicle with multiple TNF-α^+^ cells serving as positive control. **H**. Omission of the primary antibody against TNF-α verified absence of unspecific staining from the secondary antibody or DAB precipitation. **I**. IL-8 was identified in one TMT-set including five PRD patients (log2 fold change in relative expression with internal control set to 0). Lines connect the biopsy pairs

Low molecular weight cytokines with rapid mRNA turnover are not optimally detected with RNAseq or TMT mass spectrometry; accordingly, neither TNF-α mRNA nor protein were detected using the omics methods. Therefore, to validate the pathway analysis data, expression of TNF-α in the biopsied mucosa was semi-quantified with Western blot analysis (Fig. 5A). Five out of six high-dose biopsies from irradiated cancer survivors had higher mucosal concentrations of TNF-α than their internal control biopsy. TNF-α was nearly undetectable in the non-irradiated controls.

### Neuroendocrine cells contribute to TNF-α signaling in PRD

During chronic intestinal inflammation, TNF-α is believed to be produced primarily by macrophages, but also by multiple other immune cell types, as well as epithelial and stromal cells. To detect TNF-α in the biopsies, an antibody directed against TNF-α and the Novolink polymer detection system was used on paraffin sections. Multiple sparsely distributed cells in the crypt lining exhibited strong TNF-α immunoreactivity. Immunohistochemistry on adjacent sections using an antibody directed towards Chromogranin A (CgA) revealed that these cells were neuroendocrine cells (Fig. 5B-E). As expected, we also found TNF-α immunoreactivity in cells of the lamina propria (Fig. 5F), with levels varying greatly between samples. Two adjacent sections of a lymphoid follicle were used as a positive control (Fig. 5G; TNF-α primary antibody applied) and a negative control (Fig. 5H; TNF-α primary antibody omitted).

### IL-8 cytokine expression characterizes low-grade mucosal inflammation in PRD

Both TNF-α and IL-17 can stimulate the production of IL-8 which is pivotal in chronic inflammation, as it attracts neutrophils to the mucosa in a positive feedback loop (Hata et al. 2002) (Mitsuyama et al. 1994). In addition, IL-8 promotes the transition of fibroblasts to myofibroblasts and stimulates angiogenesis**.** TMT mass spectrometry revealed a multifold increase in IL-8 protein in high-dose biopsies from all five patients analyzed on one of two reagent sets (F5g. 5I). IL-8 was not detected in the remaining five subjects on the second reagent set, likely due to technical variability between the two runs.

### PRD mucosa expresses immune cell trafficking mediators targeted by IBD biologics

**Fig. 6:**
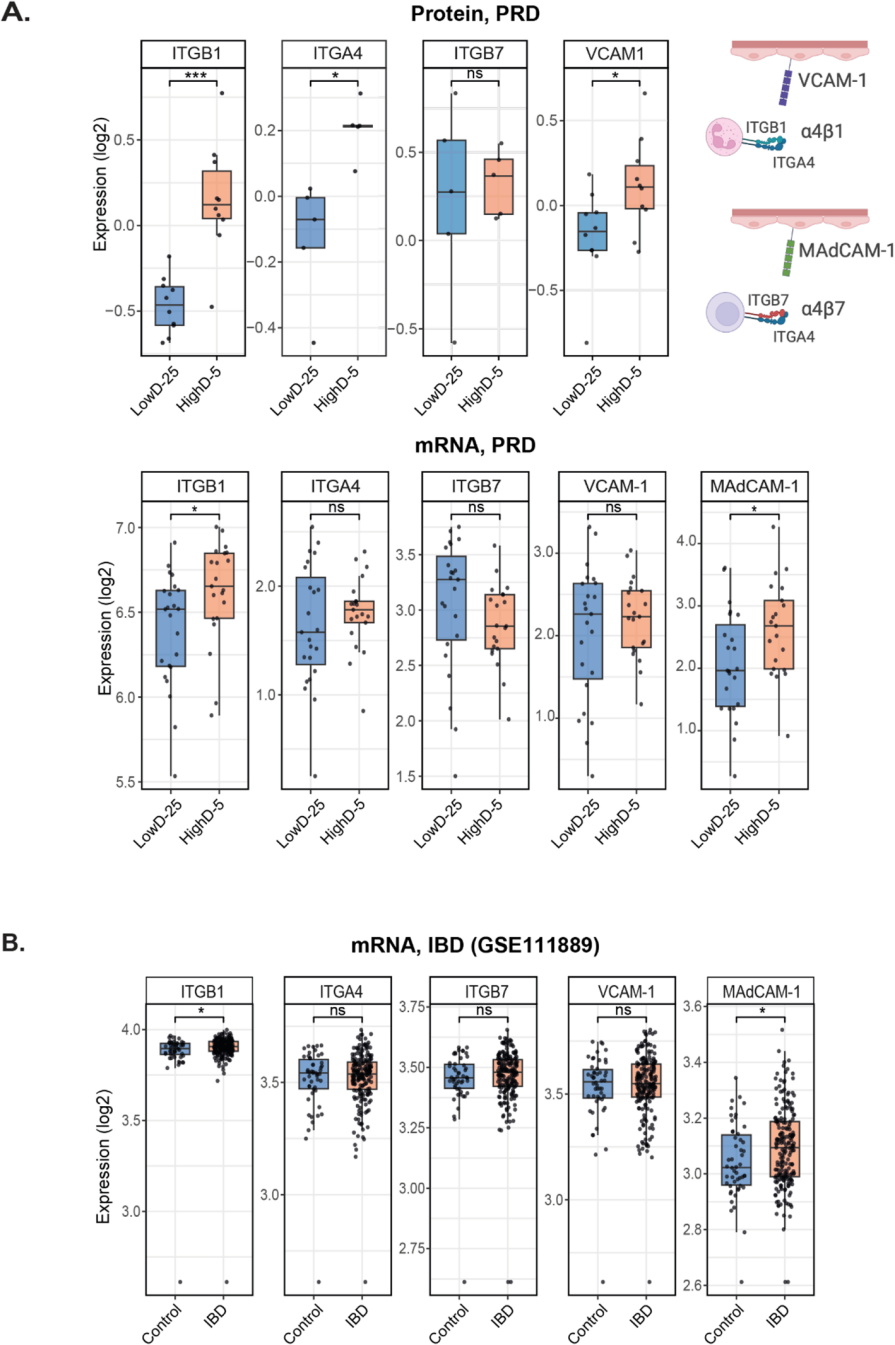
Expression of immune cell trafficking mediators in PRD and IBD. The expression of ITGB1, ITGA4, ITGB7, VCAM-1, MAdCAM-1 was assessed across three datasets. **A.** Protein expression of ITGB1, ITGA4, ITGB7, VCAM-1, MAdCAM-1 in biopsies from PRD patients were analyzed using samples obtained 25 cm from the anal verge (LowD-25, *n* = 10) and 5 cm from the anal verge (HighD-5, *n* = 10). The expression of ITGB1, ITGA4 and VCAM-1, but not ITGB7, was increased in the HighD-5 biopsies, whereas MAdCAM-1 was not detected. Bulk RNAseq analysis (LowD-25, *n* = 21; HighD-5, *n* = 21) revealed higher expression of ITGB1 and MAdCAM-1 in the HighD-5 group. **B.** Analysis of the GSE111889 RNA-seq dataset (HC, n = 50; IBD, n = 201) showed higher expression of ITGB1 and MAdCAM-1 mRNA in patients with IBD compared with healthy controls. Normalized expression values (log2-transformed) were compared between groups using the Wilcoxon rank-sum test. Statistical significance was displayed using standard significance codes: *p* < 0.05 *, p < *0.01* **, and p < *0.001* ***

TNF-α and IL-8 strongly attract circulating immune cells to the site of inflammation. In IBD, integrin inhibitors vedolizumab and natalizumab block the recruitment by targeting α4β7 integrin (vedolizumab) or the α4 integrin subunit (natalizumab) on immune cells, preventing their binding to endothelial adhesion molecules. The α4 subunit (ITGA4) pairs with ITGB1 to form α4β1, which binds endothelial adhesion molecule VCAM-1, and with ITGB7 to form α4β7, which binds endothelial adhesion molecule MAdCAM-1 (Zundler et al. 2023). We assessed the expression of all five markers—ITGA4, ITGB1, ITGB7, VCAM1, and MAdCAM1—in the IBD data sets (Crohn’s disease + ulcerative colitis; n = 201) and healthy controls (n = 50), as well as in the PRD samples from our proteomics (n = 10) and transcriptomics (n = 21) datasets (Fig. 6). Not all markers were detected in every biopsy, resulting in variable sample sizes across analyses. Both IBD and PRD biopsies had higher expression of ITGB1 and MAdCAM-1 mRNA. The translational correlation between the mRNA dataset and the protein data set in PRD was only partial, as, in addition to IGTB1, VCAM-1 and ITGA4 were elevated on protein level. MAdCAM-1 was not detected on protein level.

### High symptom intensity correlates with the expression of mRNA coding for antigen-presenting molecules

**Fig. 7:**
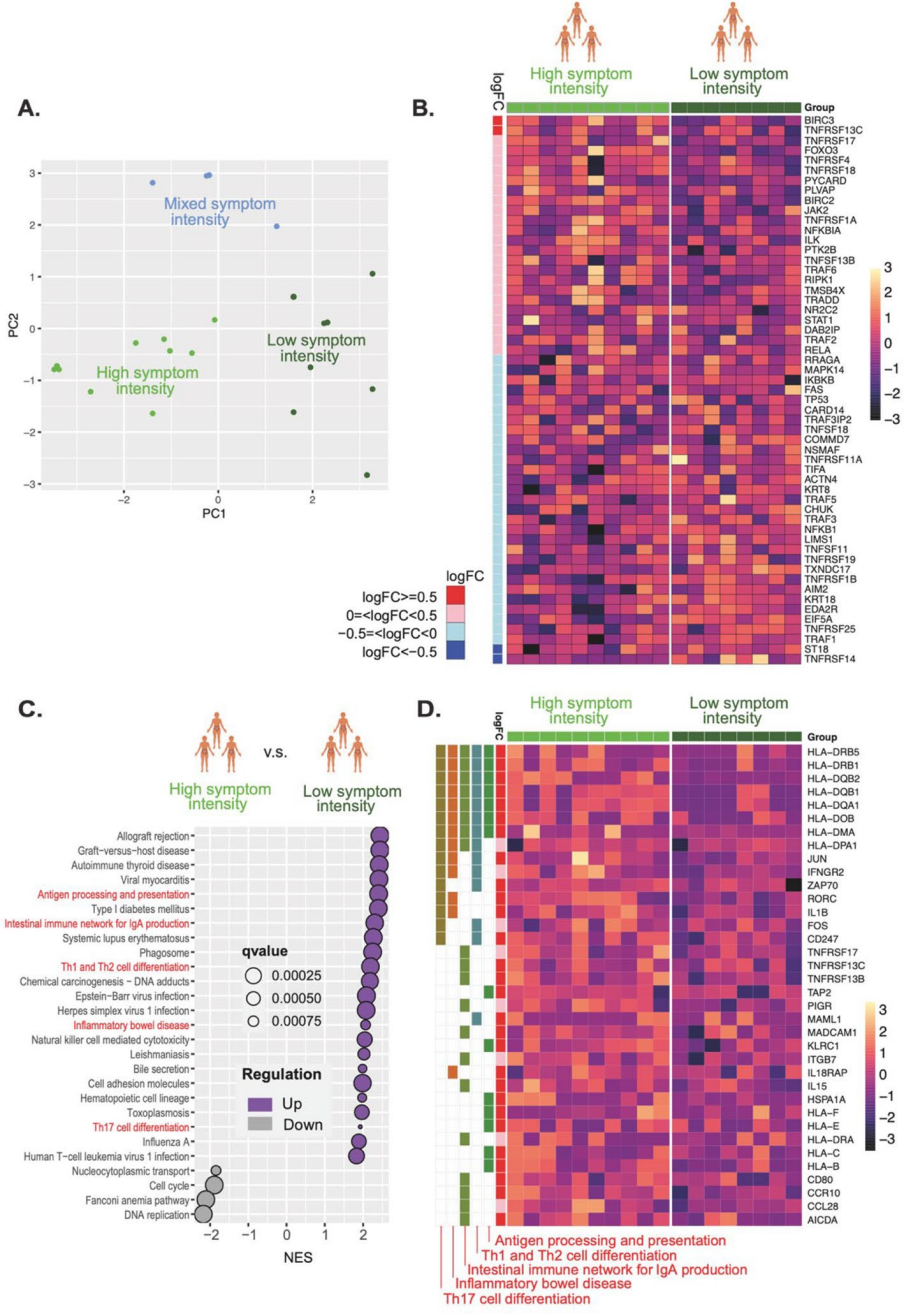
Relationship between high symptom intensity and immune activity. **A.** Clustering of PRD patients according to their symptom intensity documented with clinimetric questionnaires. Three clusters could be discerned. **B.** Heatmap showing a comparison of the expression of TNF-related genes in high-intensity and low-intensity clusters. No clear separation was found. **C.** KEGG-analysis of biological processes between the two groups revealed enrichment of several inflammatory processes in the high-intensity group. **D**. Heatmap of leading-edge gene expression in selected inflammatory processes. Gene expression encoding for multiple antigen-presenting molecules and pro-inflammatory cytokine IL1β was increased in the high-intensity group compared to the low-intensity group

To evaluate the possibility of a connection between chronic low-grade inflammation and symptom intensity, we clustered the study participants based on their symptom intensity scores retrieved from three questions related to urgency, tenesmus and loose stools in the clinimetric questionnaires (Fig. 7 A; see Additional Material 1 for questions and scoring). The participants clustered into three groups: high symptom intensity, mixed symptom intensity and low symptom intensity. We then compared the two extreme groups, high symptom intensity and low symptom intensity, with mRNA expression levels for genes known to be involved in the TNF signaling pathway (GO term GO:0033209, Fig. 7 B). We focused on mRNA only since the small sample size for protein expression limited our ability to draw meaningful conclusions from comparisons with symptom intensities. We found no evidence of a connection between TNF signaling and symptom intensity. However, a comparison between the two groups regarding biological processes revealed 23 enriched and four suppressed biological pathways (Fig. 7C). Enriched biological pathways related to inflammation were selected for deep probing of differential gene expression between the two groups. These pathways were “Antigen processing and presentation”, “Intestinal immune network for IgA production”, “Th1 and Th2 cell differentiation”, “Inflammatory bowel disease”, and “Th17 cell differentiation”. Multiple genes involved in antigen presentation showed increased expression in patients with high symptom intensity, including various HLA (human leukocyte antigen) subtypes, the co-stimulatory molecule CD80 (cluster of differentiation 80), the cytokine IL-1β, and the immune cell trafficking molecule MAdCAM-1, among others (Fig. 7D)**.** IL-1β is a potential treatment target in IBD as it promotes inflammation and fibrosis, but it was not differentially expressed in the high- versus low-dose biopsies and thus went undetected in the preceding pathway analyses.

## Discussion

By combining RNA sequencing with TMT mass spectrometry, we identified multiple pathophysiological pathways that remain active in irradiated mucosa years after pelvic radiotherapy. Metabolic programs were chronically suppressed in PRD, whereas inflammation and tissue-repair programs were enriched. Among processes similarly regulated in both IBD and PRD was TNF-α signaling through NF-κB, a central therapeutic target in IBD. We found that TNF-α is chronically expressed in the PRD mucosa of irradiated pelvic cancer survivors, and immunohistochemistry suggested that enteroendocrine cells represent a major source of this cytokine. Furthermore, PRD mucosa showed enrichment of IL-8, a key neutrophil attractant, along with immune-cell trafficking molecules that are inhibited by integrin-blocking IBD therapies. In contrast, JAK-STAT signaling—another feature targeted by IBD biologics—was observed exclusively in the IBD datasets. Moreover, there was only weak evidence of IL-6 and IL-17 expression. While we were unable to link TNF-α signaling to symptoms of PRD, irradiated pelvic cancer survivors with high symptom intensity exhibited increased expression of genes related to antigen presentation including IgA production, reflecting heightened mucosal immune cell activity.

TNF-α plays a key role in the pathogenesis of both ulcerative colitis and Crohn’s disease, mediating perturbations in the immune response that lead to chronicity. To our knowledge, the first comprehensive report on cytokine expression in the mucosa of PRD patients was published in 2019 by Reis-Ferreira et al. (Reis Ferreira et al. 2019). Although the primary focus of that study was the microbiome in PRD, the authors quantified the expression of 29 cytokines in the mucosa of nine PRD patients 2–10 years after pelvic radiotherapy for prostate cancer. The authors were unable to detect inflammation in the mucosal tissues; however, the presented data suggests increased TNF-α expression in a few subjects. Here, using multi-omics, we detected TNF-α pathway activation in high-dose irradiated biopsies, along with IL-8, and limited signs of IL-6 and IL-17 pathway activation. We chose to focus on TNF-α as this pro-inflammatory cytokine is a major target in IBD, with well-established treatments. In addition, the anti-TNF-α biologic infliximab is regularly used in the clinic to treat colitis induced by immune checkpoint inhibitors in cancer patients (Brahmer et al. 2018). Moreover, we recently published a case report on the successful treatment of severe PRD with infliximab (Toft Morén et al. 2023).

Cells in the lamina propria of PRD subjects exhibited weak but detectable immunoreactivity for TNF-α, with great variation between subjects. In contrast, CgA^+^ enteroendocrine cells in the crypt lining displayed strong and stable TNF-α immunoreactivity. Enteroendocrine cells synapse directly onto the nervous system and orchestrate the gastrointestinal response to various stimuli in the crypt microenvironment by producing a wide range of molecules, such as serotonin, glucagon-like peptides (GLPs), peptide YY, and motilin (Worthington et al. 2018). While we have been unable to find literature describing the production of TNF-α protein from enteroendocrine cells in vivo, TNF mRNA is expressed in intestinal crypts (Reyes et al. 2023) and neuroendocrine tumor cells have been reported to produce TNF-α in vitro (Bogunovic et al. 2007). Our finding suggests that enteroendocrine cells contribute significantly to TNF-α in the irradiated mucosa, possibly as a response to luminal antigens, as we have previously shown that irradiated pelvic cancer survivors display a sustained, low-level infiltration of bacteria into the mucosa (Devarakonda et al. 2023). In IBD, as in PRD, there is persistent pro-inflammatory stimulation by luminal antigens due to the disrupted mucosal barrier. While the major source of TNF-α in IBD is believed to be immune cells, circulating levels of the enteroendocrine marker CgA increases markedly in IBD patients and correlates to serum TNF-α (Sciola et al. 2009). Further investigation into the potential role of enteroendocrine cells in TNF-α-mediated mucosal inflammation could provide valuable insights into disease pathophysiology.

Stimulation of cell surface receptors, including TNFR1, by microbial components and/or pro-inflammatory cytokines such as TNF-α, triggers the canonical NF-κB-pathway. This leads to further production of proinflammatory cytokines and chemokines, along with the recruitment of immune cells. If unresolved, the release of tissue-degrading enzymes such matrix metalloproteinases (MMPs) can amplify and propagate tissue damage (Linares et al. 2021). On the other hand, TNFR2 activation of the non-canonical pathway by low levels of TNF-α, lymphotoxin from immune cells, and during epithelial stress, among others, regulates cell proliferation and survival (Souza et al. 2023; Taniguchi and Karin 2018). We found that in PRD, both the canonical and non-canonical pathways appear to be active, interconnecting inflammatory responses with regeneration and repair. The regenerative profile was unique for PRD, as processes such as hedgehog signaling and DNA repair were not found in the IBD data sets. The enrichment of mRNAs encoding DNA repair genes indicates a sustained need for active DNA repair years after completed radiotherapy. However, it is plausible that the strong suppression of metabolic programs observed in the PRD tissue impedes successful tissue repair. Nonetheless, the regenerative profile of the irradiated mucosa reveals remarkably persistent healing efforts following radiation injury, which could potentially be harnessed for therapeutic purposes.

In IBD, a key component of the inflammatory response following TNF-α binding to TNFR1 is the production of IL-8, and its expression correlates with inflammation severity (Zhu et al. 2021) (Mazzucchelli et al. 1994). IL-8 stimulates the transition of fibroblasts to myofibroblasts and recruits neutrophils in a positive feedback loop that sustains inflammation (Mitsuyama et al. 1994). IL-8 can also be induced via TNFR1-stimulated necroptosis, a caspase-independent cell death where MLKL-mediated membrane puncture releases inflammatory cell components, triggering mucosal macrophage and neutrophil activity (Akanyibah et al. 2024). Notably, a recent study published on the preprint server *BioRxiv* revealed that necroptosis was found in biopsies from “histologically non-inflamed” IBD tissue, causing epithelial cell death (Jiyi Pang (n.d.)). Thus, the expression of MLKL mRNA and protein in the high-dose irradiated biopsies combined with a multifold increase of IL-8 protein may contribute to fibrosis and interfere with tissue recovery. In line with this, interference with CXCR1 and CXCR2—the primary receptors for IL-8—as well as with their additional ligands, CXCL1 and CXCL6, all of which were upregulated in both the IBD and PRD datasets, has been shown to attenuate fibrotic responses in several animal models, including radiation-induced lung fibrosis in mice (Fox and Haston 2013). Of note, the elevated expression of additional pro-inflammatory factors, such as the tissue-degrading enzymes MMP-3 and MMP-9, have previously been proposed to reflect dynamic fibrotic tissue remodeling, rather than fixed scar tissue (Strup-Perrot et al. 2004), raising the possibility that therapeutic interference with pro-fibrotic factors may prove fruitful well beyond the acute phase of injury.

In earlier work, we demonstrated a subtle but permanent increase in neutrophil presence, along with elevated neutrophil degranulation proteins, in the irradiated mucosa of pelvic cancer survivors (Devarakonda et al. 2023). TNFα and IL-8 are potent activators of the integrin–adhesion molecule axis that mediates immune cell trafficking from the blood to the mucosa. Targeting this axis with integrin inhibitors such as vedolizumab or natalizumab is an established therapeutic strategy in IBD. We therefore investigated the mRNA and protein expression of selected adhesion molecules and integrin subunits in PRD and compared these findings with the corresponding mRNA expression in IBD datasets. Similar to IBD, expression of the α4 (ITGA4) and β1 (ITGB1) integrin subunits—as well as VCAM-1—was increased in PRD on protein level. Their expression supports continuous immune cell recruitment to the mucosa of irradiated pelvic cancer survivors through the α4β1-VCAM-1 axis, which is targeted by natalizumab in IBD. Although the MAdCAM-1 protein was not detected with TMT mass spectrometry in the PRD samples, the expression of MAdCAM-1 mRNA suggests that the α4β7–MAdCAM-1 axis is likely active as well. This is consistent with its established role in IBD, where it is targeted by both natalizumab and vedolizumab. Notably, MAdCAM-1 mRNA expression was augmented in PRD patients with high symptom intensity, compared to patients with low symptom intensity. Nevertheless, based on the current data, we cannot exclude the possibility that immune cell recruitment in PRD relies more heavily on the α4β1/VCAM-1 axis—which is broadly used across diverse immune cell types and tissue sites—than on the gut-restricted α4β7/MAdCAM-1 pathway primarily engaged by gut-homing leukocytes. If so, it may have implications for the potential efficacy of mechanistically distinct integrin-targeted therapies in PRD.

Persistent, low-grade inflammation could promote fibrosis and explain why symptoms can emerge years after completing cancer treatment and may worsen over time. Urgency, tenesmus and loose stools are very debilitating and could, at least in part, be related to inflammation, as mucosal inflammation is known to trigger these symptoms in other bowel conditions (Olesen et al. 2004; DuPont 2014). Here, we used clinimetric questionnaires to document the study participants’ symptoms at the time of biopsying and used the information to probe a possible connection between inflammatory processes and these three symptoms. While we did not find evidence of a correlation between TNF-α activity in the tissues and high symptom intensity, study participants with high symptom intensity showed enrichment in multiple inflammatory biological processes compared to those with low symptom intensity. Upon closer inspection of these processes, mRNAs coding for IL1β, MAdCAM-1, ITGB7 and eleven different HLA-proteins were increased in patients with high symptom intensity compared to those with low symptom intensity. Both mRNA coding for HLA-DQs, which present self-antigen, and HLA-DRs, which present foreign antigens, were increased. Although the comparison between symptom intensity and mRNA expression would benefit from a larger study cohort, these findings support the hypothesis that low-grade chronic inflammation following pelvic radiotherapy may promote certain radiation-induced bowel symptoms. Nonetheless, it is likely only a contributing factor. For example, poor bile acid reabsorption due to radiation-induced damage of the terminal ileum is a known cause of loose stools. Radiation-induced damage to innervation is also believed to interfere with normal sensory input and bowel movements. Such additional pathophysiological mechanisms may very well override the impact of low-grade inflammation and reduce the potential efficacy of IBD biologics in all, or at least a subset, of PRD patients. This may also help explain why anti-inflammatory agents have thus far yielded limited or no success in treating the bowel symptoms of PRD.

In this study, we employed a systematic sampling approach, including irradiated cases with internal controls as well as non-irradiated controls. The on-site biopsy collection with immediate transfer of tissue samples to optimal storage conditions provided high-quality protein and RNA samples. While this ensures data reliability and reproducibility, there is high demographic variability in the sampling population regarding pelvic cancer diagnosis, treatment regimen, and time-since-treatment. This heterogeneity may have diluted the strength of some of our findings. In addition, while our study presents evidence of multiple pathophysiological pathways including TNF-α expression and other inflammatory activity, we do not yet know whether these processes contribute to any of the symptoms reported by PRD patients, or whether the covariation of markers of antigen presentation and symptom intensity is a secondary effect. Another important question that warrants investigation is whether the chronic inflammation in PRD underlies the progression of fibrosis and prevents tissue recovery.

Our comparison of ulcerative colitis and Crohn’s disease to PRD is not intended to suggest a close relationship between the three conditions. The differences between IBD and PRD are numerous and beyond the scope of this discussion. In brief, PRD results from tissue exposure to ionizing radiation – an artificial and transient aggressor that initiates a complex cascade of tissue responses. In contrast, IBD is believed to arise from interactions between genetic and environmental factors, triggering an excessive T-cell-mediated immune response to commensal gut microbiota. In Crohn’s disease, any part of the gastrointestinal tract can be affected, while ulcerative colitis affects the colonic mucosa. In PRD, pathological changes occur mainly in the area exposed to radiation, usually the most distal part of the bowels. Moreover, while inflammation is regarded as the main culprit in IBD, PRD is believed to be characterized mainly by ischemia and fibrosis. Yet, despite the obvious differences between IBD and PRD, there are also notable similarities. Erythema, edema, bleeding, fistulation, and ulcers are found in IBD as well as PRD, and the conditions also share characteristic symptoms such as diarrhea, urgency, and tenesmus. Of particular importance may be the fact that all three conditions exhibit a disrupted intestinal barrier, which likely has profound implications for bowel health. Taken together, the extensive body of knowledge from IBD research, which far exceeds that of PRD, can provide valuable insights into understanding PRD. Moreover, it is conceivable that established or novel IBD treatments might prove beneficial for PRD, generally or in a subgroup of patients.

## Conclusions

PRD affects millions of cancer survivors worldwide and remains a major clinical challenge. By integrating advanced multi-omics approaches with standard laboratory techniques and publicly available datasets, we identified both shared and distinct mucosal inflammatory signatures between PRD and IBD. These insights may help guide the development of novel treatment approaches for PRD. If chronic low-grade mucosal inflammation contributes to the late pathophysiology of the irradiated intestinal mucosa and promotes PRD symptom development, further exploration of IBD biologics – particularly TNF-α blockers and integrin inhibitors – as therapeutic options may prove fruitful.

## Supplementary Information

Additional Material 1. Fig. S1. Comparison of activated or suppressed pathways in PRD and IBD using GSEA-KEGG pathway analysis. Regulated pathways identified from mRNA expression in PRD and the IBD data sets are depicted with circles. Diamonds depict data retrieved from the proteomic analysis. The x-axis within each of the three panels shows the normalized enrichment score. A positive value represents an enriched pathway in case biopsies versus controls, and a negative value represents a suppressed pathway. Seven biological pathways were differentially regulated between case biopsies and controls in both PRD and UC and/or CD. 57 pathways were unique for PRD, and 18 were only identified in UC and/or CD. Pathways known to be involved in the IBD inflammatory response and targeted by IBD treatments are highlighted in red. Symbol size is based on the q-value. Fig. S2. In-depth analysis of IL-17 and JAK-STAT signaling. The change in mRNA expression levels of individual genes in the A. IL-17 signaling pathway, B. IL-6 JAK-STAT3, and C. JAK-STAT signaling pathway were assessed between the three conditions and their respective controls. The top row represents the regulation of the corresponding protein in PRD, if it was identified in the TMT mass spectrometry analysis. Only the leading-edge genes that were significantly differentially expressed in at least one group are shown. White fields: NA/non-significant. [FDR<0.001 for transcriptomics and 0.05 for proteomics, |log2FC|>0.5]). Fig. S3. Regulation of TNF Signaling Genes in IBD and PRD. Pathview visualization of all genes, regardless of the p-value, with a logFC value >1 identified in the TNF pathway for A. PRD, B. UC, and C. CD. The color key represents fold change from the control, where green is downregulated and red is upregulated. |logFC|<1 is set to 0. logFC values beyond [-4,4] are set to -4/4. Fig.S4. Regulation of IL-17 Signaling Genes in IBD and PRD. Pathview visualization of all genes, regardless of the p-value, with a logFC value >1 identified in the IL-17 pathway for A. PRD, B. UC, and C. CD. The color key represents fold change from the control, where green is downregulated and red is upregulated. |logFC|<1 is set to 0. logFC values beyond [-4,4] are set to -4/4. Fig. S5. Regulation of cytokine-cytokine receptor interaction genes in IBD and PRD. Pathview visualization of all genes, regardless of the p-value, with a logFC value >1 identified for cytokine-cytokine receptor interaction in A. PRD, B. UC, and C. CD. The color key represents fold change from the control, where green is downregulated and red is upregulated. |logFC|<1 is set to 0. logFC values beyond [-4,4] are set to -4/4.

Additional Material 2.

## Data Availability

The datasets analyzed during the current study are available from the corresponding author on reasonable request.
