# Supplementary material for: TNFα signaling in radiation-induced chronic bowel dysfunction suggests therapeutic potential for IBD biologics
Source: Mol Med. 2026 Apr 9;32:53. doi: 10.1186/s10020-026-01441-4 (PMC13064405; doi:10.1186/s10020-026-01441-4)

Supplementary Figure 1

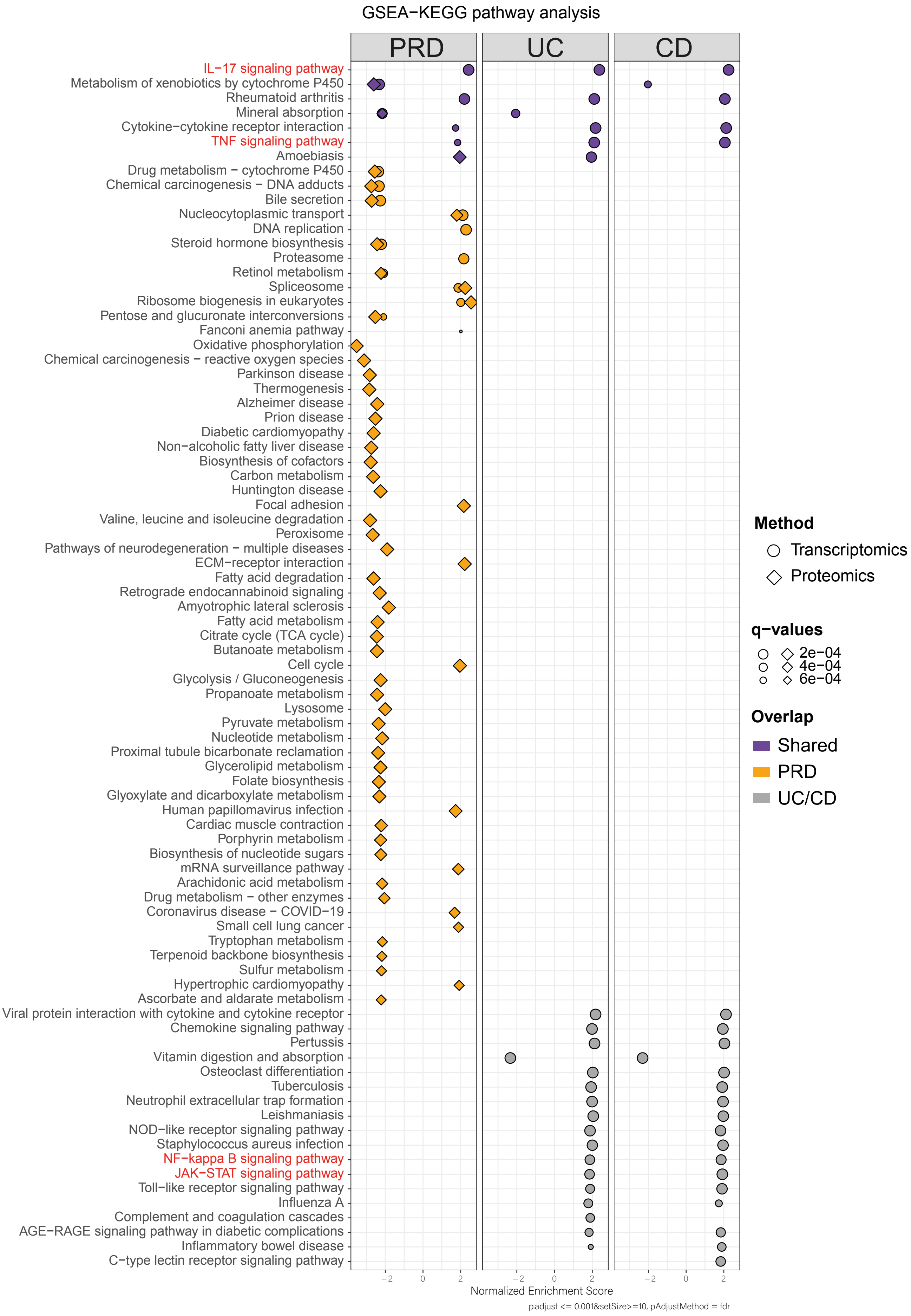

Supplementary Figure 2

A. IL−17 signaling pathway

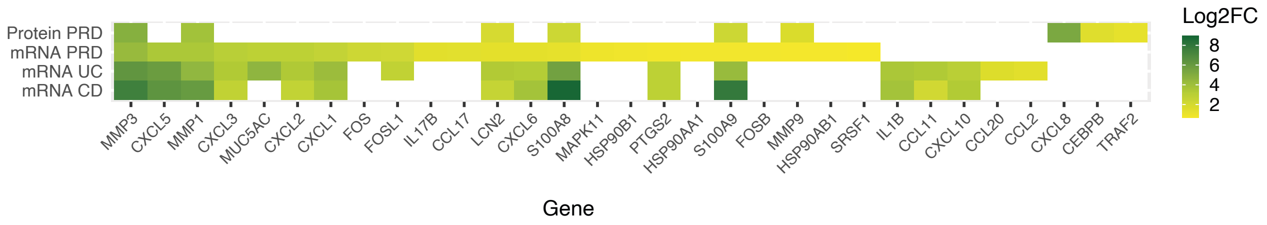

B. IL6 JAK-STAT3 signaling pathway

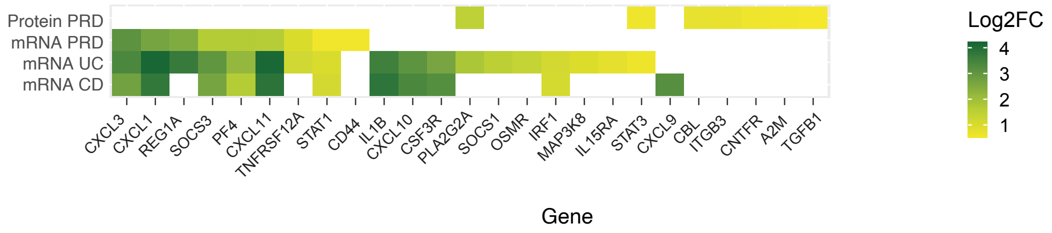

C. JAK-STAT signaling pathway

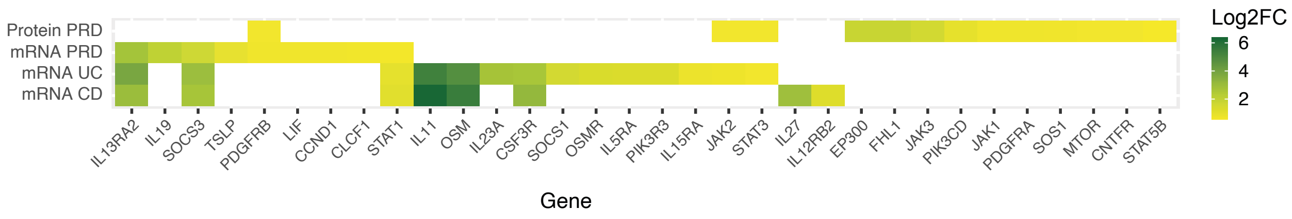

Only leading edge genes sig. in at least one group are shown (white:NA/non−significant leading edge genes) [FDR<0.001(transcriptomics)/0.05(proteomics), |log2FC>0.5]

Supplementary Figure 3

A. TNF signaling pathway, PRD

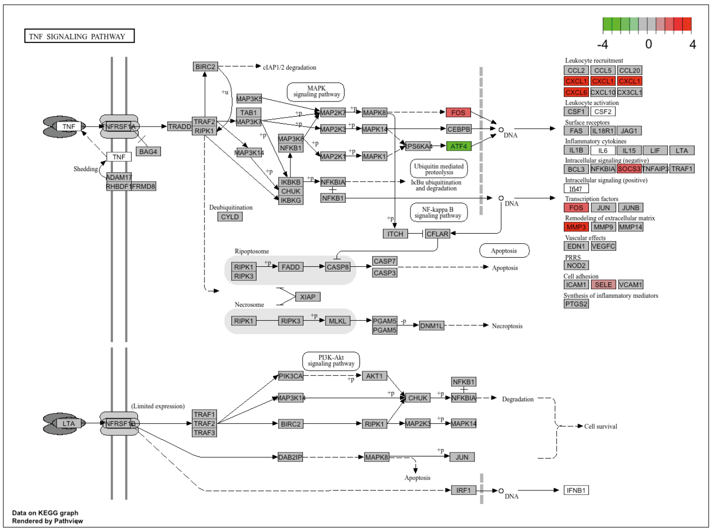

|logFC|<1=0

B. TNF signaling pathway, UC

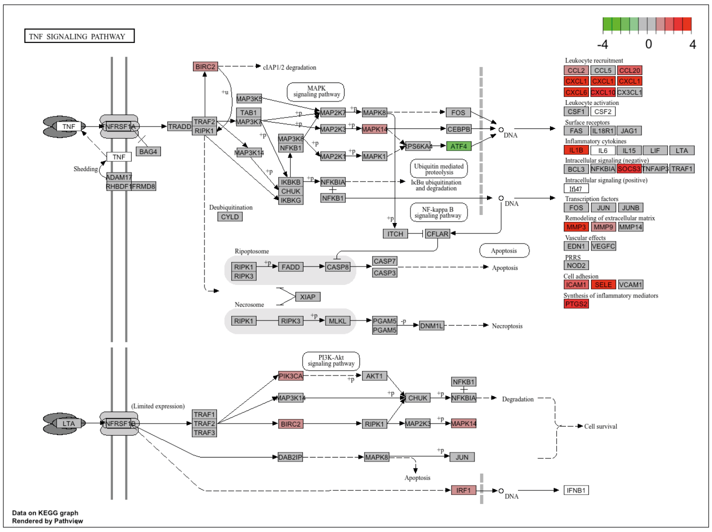

C. TNF signaling pathway, CD

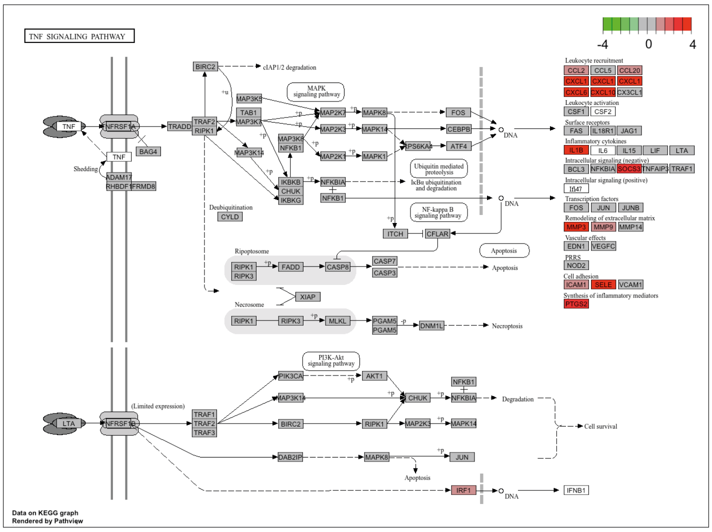

Supplementary Figure 4

A. IL-17 signaling pathway, PRD

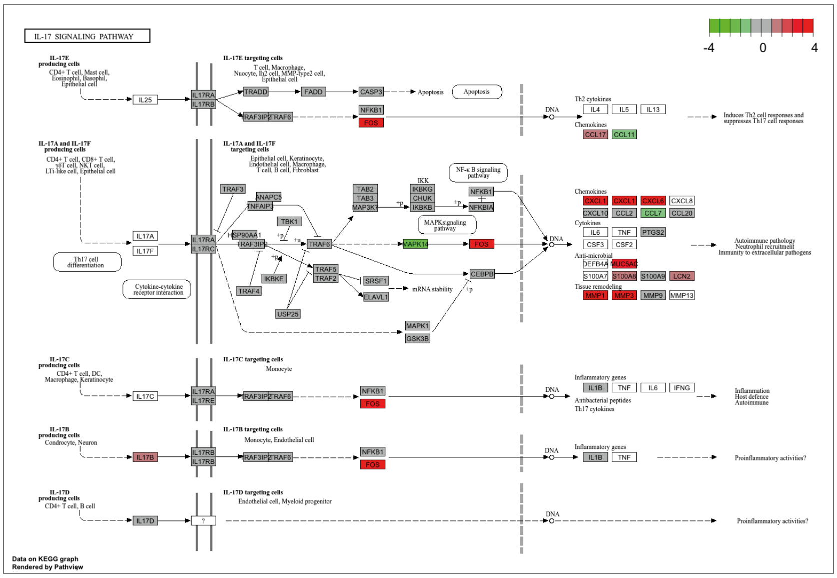

|logFC|<1=0

B. IL-17 signaling pathway, UC

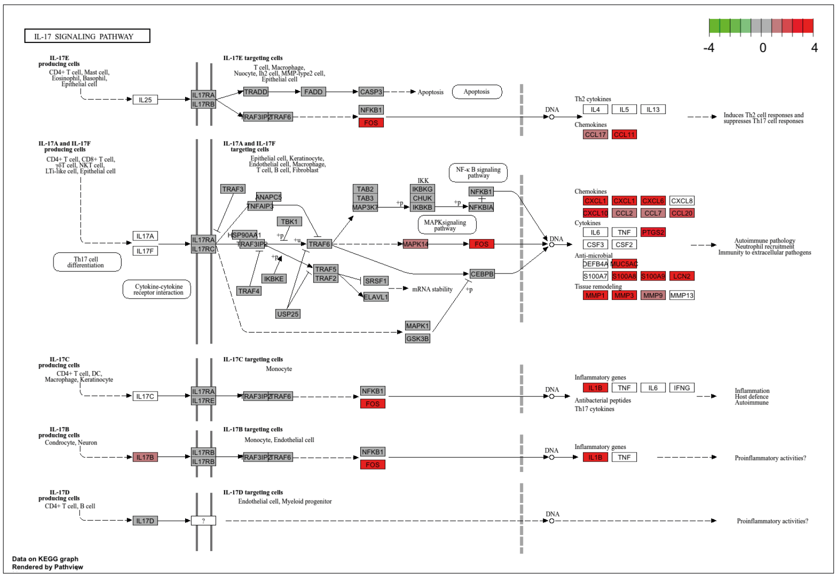

C. IL-17 signaling pathway, CD

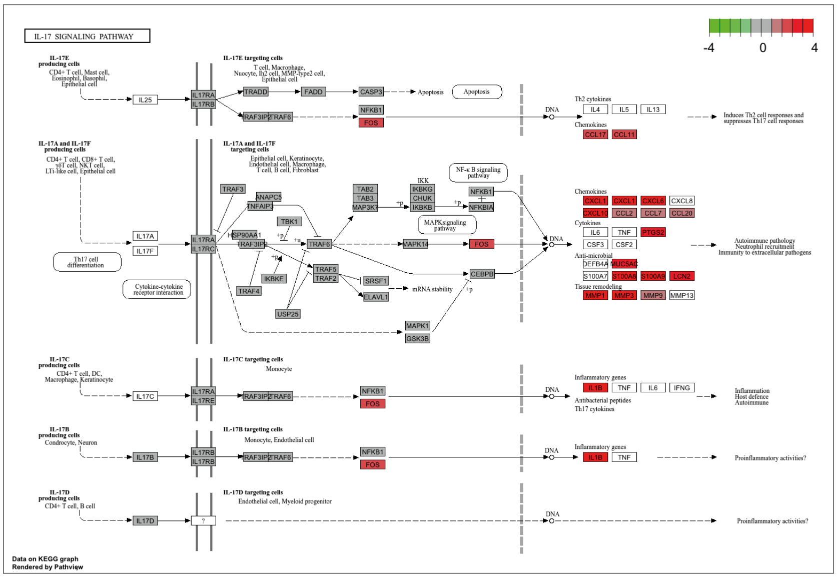

Supplementary Figure 5

A. Cytokine-cytokine receptor interaction, PRD

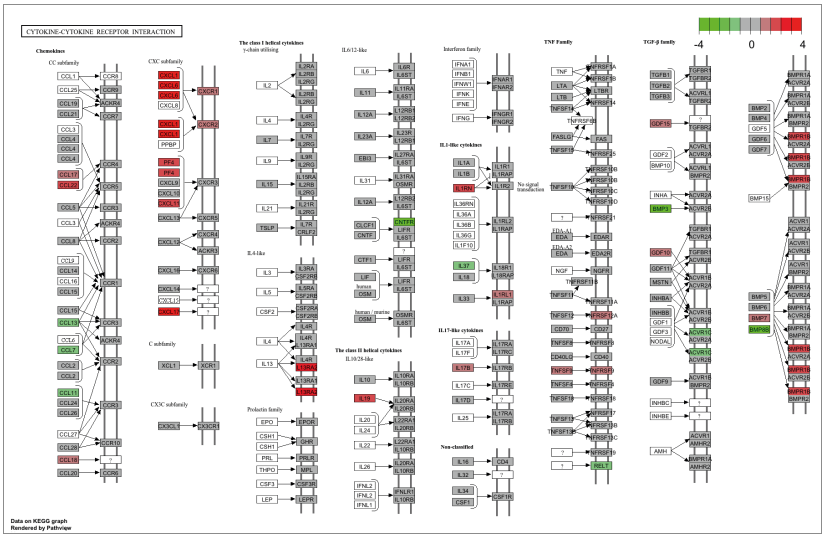

|logFC|<1=0

B. Cytokine-cytokine receptor interaction, UC

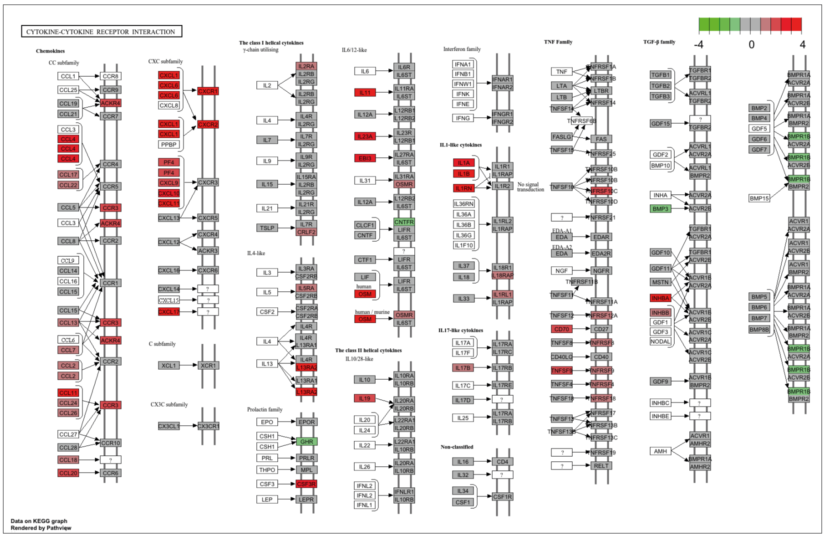

C. Cytokine-cytokine receptor interaction, CD

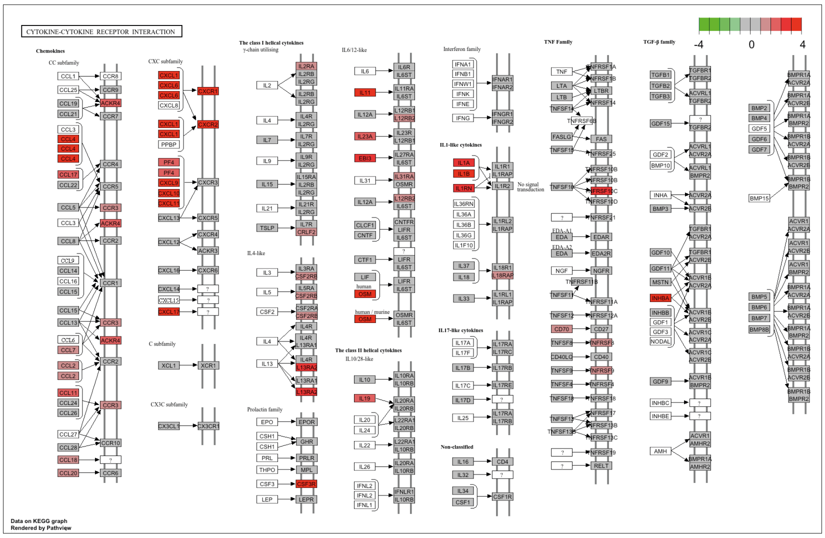

Supplement: Supplementary file 1 — Additional Material 1. Fig. S1. Comparison of activated or suppressed pathways in PRD and IBD using GSEA-KEGG pathway analysis. Regulated pathways identified from mRNA expression in PRD and the IBD data sets are depicted with circles. Diamonds depict data retrieved from the proteomic analysis. The x-axis within each of the three panels shows the normalized enrichment score. A positive value represents an enriched pathway in case biopsies versus controls, and a negative value represents a suppressed pathway. Seven biological pathways were differentially regulated between case biopsies and controls in both PRD and UC and/or CD. 57 pathways were unique for PRD, and 18 were only identified in UC and/or CD. Pathways known to be involved in the IBD inflammatory response and targeted by IBD treatments are highlighted in red. Symbol size is based on the q-value. Fig. S2. In-depth analysis of IL-17 and JAK-STAT signaling. The change in mRNA expression levels of individual genes in the A. IL-17 signaling pathway, B. IL-6 JAK-STAT3, and C. JAK-STAT signaling pathway were assessed between the three conditions and their respective controls. The top row represents the regulation of the corresponding protein in PRD, if it was identified in the TMT mass spectrometry analysis. Only the leading-edge genes that were significantly differentially expressed in at least one group are shown. White fields: NA/non-significant. [FDR<0.001 for transcriptomics and 0.05 for proteomics, |log2FC|>0.5]). Fig. S3. Regulation of TNF Signaling Genes in IBD and PRD. Pathview visualization of all genes, regardless of the p-value, with a logFC value >1 identified in the TNF pathway for A. PRD, B. UC, and C. CD. The color key represents fold change from the control, where green is downregulated and red is upregulated. |logFC|<1 is set to 0. logFC values beyond [-4,4] are set to -4/4. Fig.S4. Regulation of IL-17 Signaling Genes in IBD and PRD. Pathview visualization of all genes, regardless of the p-value, [file 10020_2026_1441_MOESM1_ESM.pdf]
