# Supplementary material for: TNFα signaling in radiation-induced chronic bowel dysfunction suggests therapeutic potential for IBD biologics
Source: Mol Med. 2026 Apr 9;32:53. doi: 10.1186/s10020-026-01441-4 (PMC13064405; doi:10.1186/s10020-026-01441-4)
Supplement: Supplementary file 2 — Additional Material 2. [file 10020_2026_1441_MOESM2_ESM.docx]

**Appendix 1**

Study participants were asked to select the response option that best reflected their experience. Scores for symptom intensity ranged from 1 to 6, with “No” scored as 1 and “Yes, about once a day“ scored as 6. The questions were (translated from Swedish):

1. In the past six months, have you experienced sudden urges to defecate requiring immediate access to a toilet?

- No
- Yes, on a few occasions
- Yes, about once a month
- Yes, about once a week
- Yes, about three times a week
- Yes, about once a day

2. In the past six months, have you experienced loose stools?

- No
- Yes, on a few occasions
- Yes, about once a month
- Yes, about once a week
- Yes, about three times a week
- Yes, about once a day

3. In the past six months, have you returned to the toilet within an hour after a bowel movement to empty your bowels again?

- No
- Yes, on a few occasions
- Yes, about once a month
- Yes, about once a week
- Yes, about three times a week
- Yes, about once a day
